# The elimination of human African trypanosomiasis: Achievements in relation to WHO road map targets for 2020

**DOI:** 10.1371/journal.pntd.0010047

**Published:** 2022-01-18

**Authors:** Jose R. Franco, Giuliano Cecchi, Massimo Paone, Abdoulaye Diarra, Lise Grout, Augustin Kadima Ebeja, Pere P. Simarro, Weining Zhao, Daniel Argaw

**Affiliations:** 1 World Health Organization, Control of Neglected Tropical Diseases, Prevention Treatment and Care, Geneva, Switzerland; 2 Food and Agriculture Organization of the United Nations, Animal Production and Health Division, Rome, Italy; 3 World Health Organization, Regional Office for Africa, Communicable Disease Unit, Brazzaville, Congo; 4 Consultant, World Health Organization, Control of Neglected Tropical Diseases, Innovative and Intensified Disease Management, Geneva, Switzerland; Institute of Tropical Medicine, BELGIUM

## Abstract

**Background:**

In the 20th century, epidemics of human African trypanosomiasis (HAT) ravaged communities in a number of African countries. The latest surge in disease transmission was recorded in the late 1990s, with more than 35,000 cases reported annually in 1997 and 1998. In 2013, after more than a decade of sustained control efforts and steady progress, the World Health Assembly resolved to target the elimination of HAT as a public health problem by 2020. We report here on recent progress towards this goal.

**Methodology/principal findings:**

With 992 and 663 cases reported in 2019 and 2020 respectively, the first global target was amply achieved (i.e. fewer than 2,000 HAT cases/year). Areas at moderate or higher risk of HAT, where more than 1 case/10,000 people/year are reported, shrunk to 120,000 km^2^ for the five-year period 2016–2020. This reduction of 83% from the 2000–2004 baseline (i.e. 709,000 km^2^) is slightly below the target (i.e. 90% reduction). As a result, the second global target for HAT elimination as a public health problem cannot be considered fully achieved yet. The number of health facilities able to diagnose and treat HAT expanded (+9.6% compared to a 2019 survey), thus reinforcing the capacity for passive detection and improving epidemiological knowledge of the disease. Active surveillance for gambiense HAT was sustained. In particular, 2.8 million people were actively screened in 2019 and 1.6 million in 2020, the decrease in 2020 being mainly caused by COVID-19-related restrictions. Togo and Côte d’Ivoire were the first countries to be validated for achieving elimination of HAT as a public health problem at the national level; applications from three additional countries are under review by the World Health Organization (WHO).

**Conclusions/significance:**

The steady progress towards the elimination of HAT is a testament to the power of multi-stakeholder commitment and coordination. At the end of 2020, the World Health Assembly endorsed a new road map for 2021–2030 that set new bold targets for neglected tropical diseases. While rhodesiense HAT remains among the diseases targeted for elimination as a public health problem, gambiense HAT is targeted for elimination of transmission. The goal for gambiense HAT is expected to be particularly arduous, as it might be hindered by cryptic reservoirs and a number of other challenges (e.g. further integration of HAT surveillance and control into national health systems, availability of skilled health care workers, development of more effective and adapted tools, and funding for and coordination of elimination efforts).

## Author summary

Human African trypanosomiasis (HAT) is a lethal neglected tropical disease (NTD) transmitted by the bite of infected tsetse flies. The disease is also known as “sleeping sickness”. During the 20^th^ century it caused enormous suffering in the endemic areas in sub-Saharan Africa. HAT transmission last soared in the late 1990s, triggering a renewed, coordinated and very successful control effort. In this paper, we present achievements towards HAT elimination, with a focus on the WHO road map targets for 2020. In particular, reported cases continue to decline, from over 30,000 cases per year at the turn of the century to 663 cases in 2020. Despite the impact of the COVID-19 pandemic, HAT surveillance was largely sustained, and the network of health facilities able to diagnose and treat the disease further expanded. Looking to the future, the World Health Organization (WHO) set bold new targets for HAT in its 2021–2030 road map for NTDs, namely: the elimination of transmission of gambiense HAT, which occurs in western and central Africa, and the elimination as a public health problem of rhodesiense HAT, which is found in eastern and southern Africa. The strong commitment of national health authorities and the international community will be essential if these goals are to be achieved.

## Introduction

Two subspecies of the unicellular flagellate parasite *Trypanosoma brucei* (i.e. *T*. *b*. *gambiense* and *T*. *b*. *rhodesiense*) are the causative agents of human African trypanosomiasis (HAT), also known as “sleeping sickness” [1]. The disease is transmitted in sub-Saharan Africa by the bite of infected tsetse flies (Genus: *Glossina*), within fairly well-delineated foci of endemicity [2–4]. Of the two recognized forms of HAT, that caused by *T*. *b*. *gambiense* is endemic in western and central Africa, whereas that caused by *T*. *b*. *rhodesiense* occurs in eastern and southern Africa [3,5]. HAT cases are also detected among travellers, tourists and migrants outside the endemic countries of sub-Saharan Africa, including in the Americas, Europe and Asia [6].

Gambiense HAT is responsible for the vast majority of reported cases [7], and it is considered mainly anthroponotic [8]. Conversely, rhodesiense HAT is more clearly a zoonosis, with reservoirs in both wildlife and livestock [9,10]. In addition to their distinctive geographical and epidemiological features, the two forms also differ in their onset and progression, with rhodesiense HAT progressing much faster than gambiense HAT [11,12]. If not correctly diagnosed and properly treated, sleeping sickness is almost invariably fatal [13].

Because it mostly affects rural, resource-poor communities in sub-Saharan Africa, HAT is considered a neglected tropical disease (NTD) [14]. During the 20th century, what has been dubbed as the “colonial disease” [15] was responsible for a number of devastating epidemics [16,17]. The latest upsurge in infections peaked in the late 1990s [18,19], when over 25,000 cases were being reported annually. Specifically, more than 35,000 cases per year were reported in 1997 and 1998 [9], and as many as 300,000 real cases were estimated [2]. To stem the rising tide, a broad alliance of stakeholders was mobilized [9]. The National Sleeping Sickness Control Programmes (NSSCPs) in the endemic countries stood at the forefront. Under the aegis and coordination of the World Health Organization (WHO), NSSCPs were supported by pharmaceutical companies, bilateral cooperation agencies, nongovernmental organizations (NGOs), research institutions and philanthropic organizations [20,21]. This collective effort succeeded in dramatically reversing the epidemiological trend and, within a decade, had brought the disease under control [9].

In 2012, WHO developed a road map on NTDs [14], which was adopted by the 66^th^ World Health Assembly in 2013. For HAT, the road map set “elimination as a public health problem” as the goal for 2020. This was subsequently defined as “fewer than 2,000 cases reported per year”, and “a 90% reduction in the areas at moderate or higher risk compared to the 2000–2004 baseline” [7]. Since the global goal was set, progress has been steady and substantial, and biennial reports showed the process of HAT elimination to be on track [7,22–24]. By 2017, fewer than 2000 cases/year were already being reported globally, with 977 reported in 2018 [7]. The progress over the past two decades has been achieved mainly through a combination of active and passive case-finding, and the treatment of individuals with detected infections. In the past few years, vector control has also contributed to curbing transmission in a few areas [1,7].

In this paper we present the latest situational update on sleeping sickness in Africa, with a focus on the period 2019–2020, review progress towards the WHO road map targets for 2020 and provide an update on the elimination of HAT at the national level. Finally, we discuss the findings in relation to future elimination goals.

## Materials and methods

### Ethics statement

This paper does not involve research with human participants. No individual data is used. All the data used are provided as epidemiological information and are fully anonymized before transmission.

### HAT elimination as a public health problem at the global level

All data presented in this paper and used to monitor HAT elimination were reported to WHO by NSSCPs, NGOs and research institutions. Data were harmonized, verified and included in the Atlas of HAT [3], a WHO initiative jointly implemented with the Food and Agriculture Organization of the United Nations (FAO) in the framework of the Programme against African Trypanosomosis [25].

The first global indicator of HAT elimination as a public health problem is the total number of cases reported annually. The target for 2020 was set as “fewer than 2,000 cases/year”. The second indicator is areas at moderate, high or very high risk of HAT. This includes areas reporting ≥ 1 case/10,000 people/year, as averaged over a five-year period. The method used to estimate the area at risk has been described in detail in previous publications [4,22]. The set target for 2016–2020 was “a reduction of 90% from the 2000–2004 baseline of 709,000 km^2^” [7,26].

In addition to the two main indicators, for which quantitative targets were defined, three secondary indicators were also routinely monitored: (i) the geographical distribution of the disease, (ii) the number of people at risk of the disease by risk category, and (iii) the coverage of the at-risk populations by control and surveillance activities. The latter is particularly challenging to estimate across Africa, and simplified proxies are currently in use. In particular, for active screening, the number of people tested and their geographical distribution is monitored [23,24]; for passive surveillance, we survey and map health facilities with capacity for diagnosis and treatment of HAT, and subsequently estimate their potential accessibility for populations at risk [27].

The list of fixed health facilities with capacities for HAT diagnosis and treatment was last compiled by WHO in 2019, using data provided by NSSCPs [7]. In this paper we present the results of the latest survey, which was carried out between February and June 2021. Finally, we provide an overview of vector control activities against HAT as implemented by NSSCPs and their partners.

### HAT elimination as a public health problem at the national level

At the national level, HAT elimination as a public health problem requires that fewer than 1 case/10,000 inhabitants/year (averaged over a five-year period) be reported from each health district [7]. This quantitative indicator must be complemented by a qualitative evaluation of control and surveillance activities at the national level, including active and passive case-finding, case management and vector control. Based on their intensity and effectiveness, these activities are categorized as “adequate”, “insufficient” or “absent”; attainment of an “adequate” level is required [7]. Countries that fulfil both criteria are eligible to apply for the validation of HAT elimination as a public health problem at the national level. To this end, the national Ministry of Health should prepare and submit a dossier to WHO for evaluation by an independent, ad hoc panel of experts. In the present paper we report on countries that have already obtained and those that have applied for WHO validation.

## Results

### Number of HAT cases reported annually

**Fig 1:**
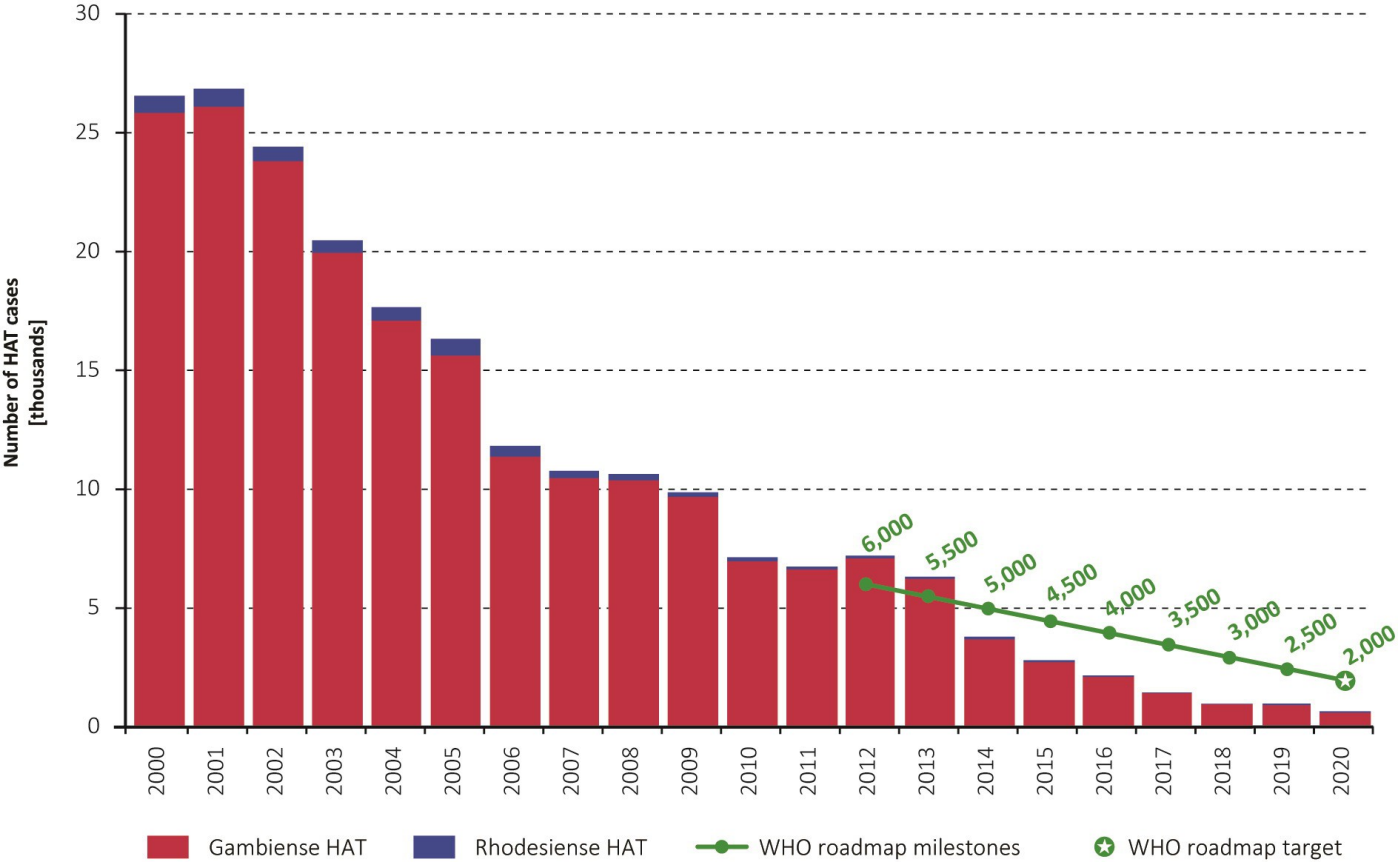
Total number of reported cases of HAT (gambiense and rhodesiense) per year (2000–2020). The green line and the green circles show the milestones and target set in the WHO road map for HAT elimination [14].

Tables 1 and 2 show the number of gambiense and rhodesiense HAT cases reported by endemic countries during 2011–2020. Fig 1 shows the overall trend in reported HAT cases since 2000, including the milestones and target that were defined for HAT elimination as a public health problem. For both forms of the disease, 992 cases were reported in 2019 and 663 in 2020.

**Table 1:** *T*. *b*. *gambiense* HAT: new cases reported between 2011 and 2020.

Country	2011	2012	2013	2014	2015	2016	2017	2018	2019	2020	Total
Angola	154	70	69	36	35	20	18	79	30	33	544
Burkina Faso	0	0	0	0	1	0	0	0	0	0	1
Cameroon	15	7	6	7	6	6	5	7	20	2	81
Central African Republic	132	381	59	194	147	101	76	57	86	39	1,272
Chad	276	197	195	95	67	54	28	12	16	17	957
Congo	61	39	20	21	36	18	15	24	17	15	266
Côte d’Ivoire	10	9	7	6	3	0	3	2	1	0	41
Democratic Republic of the Congo	5,590	5,969	5,649	3,205	2,347	1,768	1,100	660	613	395	27,296
Equatorial Guinea	1	2	3	0	0	3	4	4	3	1	21
Gabon	17	9	17	10	9	10	9	16	8	11	116
Ghana	0	0	1	0	0	0	0	0	0	0	1
Guinea	57	70	78	33	29	108	139	74	69	36	693
Nigeria	3	2	0	0	0	1	0	0	0	0	6
South Sudan	272	317	117	63	45	17	12	17	11	15	886
Uganda	44	20	9	9	4	4	0	1	2	1	94
Total	6,632	7,092	6,230	3,679	2,729	2,110	1,409	953	876	565	32,275

Other historically *T*. *b*. *gambiense* HAT endemic countries not reporting cases but with continuing surveillance activities are Benin, Mali and Togo, with occasional surveillance activities are Guinea-Bissau, Liberia, Niger, Senegal and Sierra Leone. In the Gambia no cases are reported but no surveillance activity is known.

**Table 2:** *T*. *b*. *rhodesiense* HAT: new cases reported between 2011 and 2020.

Country	2011	2012	2013	2014	2015	2016	2017	2018	2019	2020	Total
Kenya	0	2	0	0	0	0	0	0	0	0	2
Malawi	23	18	35	32	30	35	7	15	91	89	375
Uganda	84	71	43	70	28	10	13	4	5	2	330
United Republic of Tanzania	1	4	2	1	2	4	3	0	3	1	21
Zambia	3	6	6	12	9	4	3	5	15	6	69
Zimbabwe	4	9	1	3	3	1	1	0	2	0	24
Total	115	110	87	118	72	54	27	24	116	98	821

Other historically *T*. *b*. *rhodesiense* HAT endemic countries not reporting cases are Burundi, Ethiopia, Mozambique and Rwanda. Botswana, Namibia and Eswatini are considered free of the vector for the transmission of *T*. *b*. *rhodesiense* HAT [28–31].

In the past two years (2019–2020), 1,441 cases of *T*. *b*. *gambiense* were reported, corresponding to 87% of all HAT infections. Annually, this represents a 39% reduction from the previous biennium (2017–2018), and a 97% reduction from 2000–2001. Nationally, 1,008 cases were reported from the Democratic Republic of the Congo in 2019–2020, corresponding to 70% of all gambiense HAT cases. The overall intensity of active surveillance for gambiense HAT was sustained at similar levels to previous years, with 2.8 and 1.6 million people screened in 2019 and 2020 respectively, against an average of 2.4 million/year during 2000–2018. The unusual decrease in the number of people actively screened during the past year was mainly caused by the interruption for several months of active screening activities in many countries due to the pandemic of Coronavirus disease (COVID-19).

For rhodesiense HAT, 116 and 98 cases were reported in 2019 and 2020 respectively, representing a more than fourfold increase from the previous biennium that resulted from an outbreak in Malawi at the end of 2019. Despite this surge, the numbers of cases of rhodesiense HAT in 2019–2020 are still 85% fewer than those reported in 2000–2001.

Seven cases of rhodesiense HAT and 3 cases of gambiense HAT were reported from nonendemic countries in 2019–2020. These “exported” cases were detected in South Africa (5), the USA (2), Sweden (1), Germany (1) and India (1); based on their case histories, they were probably infected in Malawi (3), Zambia (2), Cameroon (2), Uganda (2) and Guinea (1). Exported cases are included in Tables 1 and 2.

### Geographical distribution of HAT

**Fig 2:**
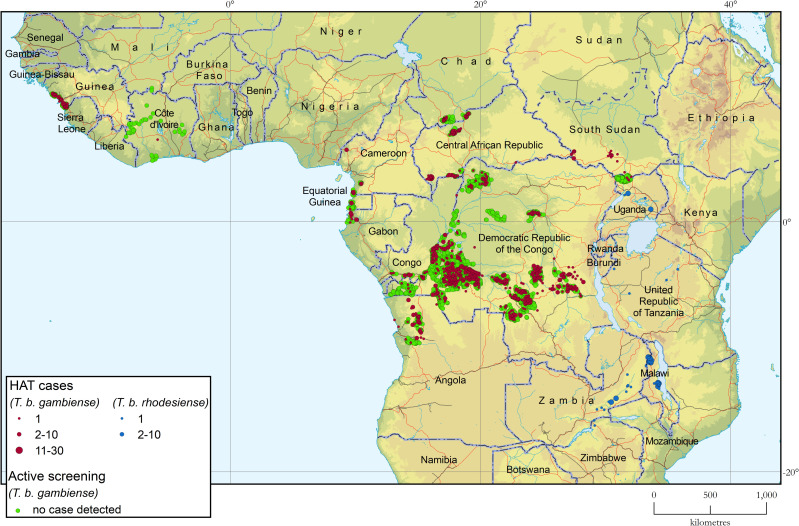
Geographical distribution of human African trypanosomiasis. Period 2019–2020. The base layers used in this map are the FAO Global Administrative Unit Layers (GAUL), Shuttle Radar Topography Mission (SRTM), FAO Inland water bodies in Africa, FAO Rivers of Africa and Vector Map Level 0 (VMap0).

Fig 2 shows the geographical distribution of sleeping sickness cases in Africa during 2019–2020. Green dots mark the location of active screening activities where no cases were detected. S1 File provides larger-scale subregional maps.

#### Gambiense HAT

In western Africa, almost all HAT cases are reported from the coastal region of Guinea (105), with only one additional case detected in Côte d’Ivoire. In Guinea, the number of cases detected is showing a sizable decrease after the resurgence in 2016–2017 following the stall in surveillance for the Ebola epidemic; control and surveillance activities were reinforced, and the coverage of populations at risk increased. In Côte d’Ivoire, one single case of HAT was reported in 2019 and none was detected in 2020, despite an extensive surveillance programme. No other country in the region reported cases during 2019–2020. Benin, Burkina Faso, Ghana, Mali and Togo maintained operational HAT surveillance, while in Nigeria surveillance activities were limited. During the same period, no surveillance and control activities were reported from the Gambia, Guinea-Bissau, Senegal, Sierra Leone and Niger.

In central Africa, HAT control and surveillance is routinely carried out in Cameroon, Central African Republic, Chad, Congo, Equatorial Guinea and Gabon. In Cameroon, the number of reported cases has been fluctuating for a few years, following variations in the intensity of control and surveillance activities. For example, these activities were reinforced in 2019 but were reduced in 2020. In Equatorial Guinea, the number of reported cases has remained stable and in the low single-digit range for over 10 years. In Chad, the number of cases has remained very low, with a further decrease in the Mandoul focus and a slight increase in the Maro focus. In the Central African Republic, improved security in 2019 allowed control activities to be intensified and more cases to be detected. Unfortunately, a reversal was observed in 2020, when the deteriorating security situation and the COVID-19 pandemic hampered access to HAT endemic areas and resulted in a decrease in case detection. In the Congo and Gabon, the number of reported cases remained stable in 2019–2020, against a backdrop of limited control and surveillance activities.

In Uganda, very few cases of gambiense HAT continue to be diagnosed, and control and surveillance measures remained robust across the endemic region of West Nile; only three autochthonous cases were reported during 2019–2020. In South Sudan, the number of reported cases remained stable and low, but control and surveillance activities were weak and hampered by civil strife. In Angola, the number of cases remained low, despite a substantial reinforcement of passive surveillance.

The Democratic Republic of the Congo remains the country with the heaviest burden of gambiense HAT, even though a sizable reduction in detected cases was reported in 2020. However, the latter reduction must be interpreted in the context of reduced surveillance caused by COVID-19.

#### Rhodesiense HAT

The number of reported cases of rhodesiense HAT soared in 2019 (116 cases). This was mostly due to an outbreak in Malawi, which was responsible for 78% of the cases declared that year, and, to a lesser extent, an increased number of cases in Zambia. Both the outbreak in Malawi and the increase in case numbers in Zambia abated in 2020. Other countries endemic for rhodesiense HAT (Uganda, United Republic of Tanzania and Zimbabwe) continue to declare only sporadic cases, while Kenya and Rwanda, with a surveillance system in place, continue to report none. The majority of rhodesiense HAT cases currently being detected in eastern and southern Africa are linked to protected areas and to the wildlife reservoir. However, diagnostic capacities remain weak in most endemic countries, which entails a non-negligible albeit difficult-to-estimate level of under detection. The number of cases of rhodesiense HAT diagnosed in nonendemic countries remains low and with a somewhat downward trend.

### Areas and population at risk of HAT

**Fig 3:**
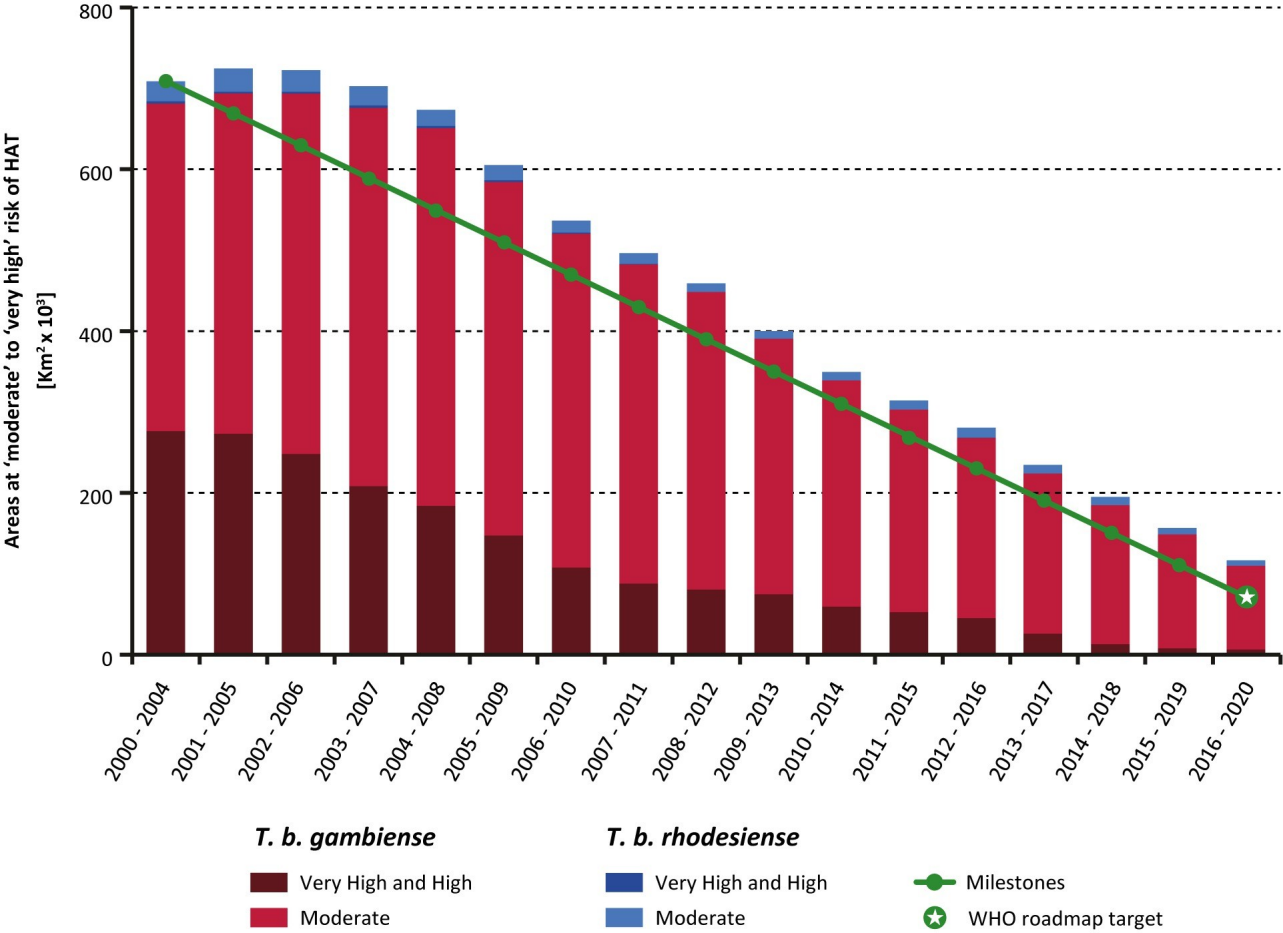
Trends in areas at risk of gambiense and rhodesiense HAT where the disease is still considered as a public health problem (2000–2004 to 2016–2020). The green line and green circles show the milestones and target set by the WHO Strategic and Advisory Group for Neglected Tropical Diseases to achieve the elimination of HAT as a public health problem by 2020.

**Fig 4:**
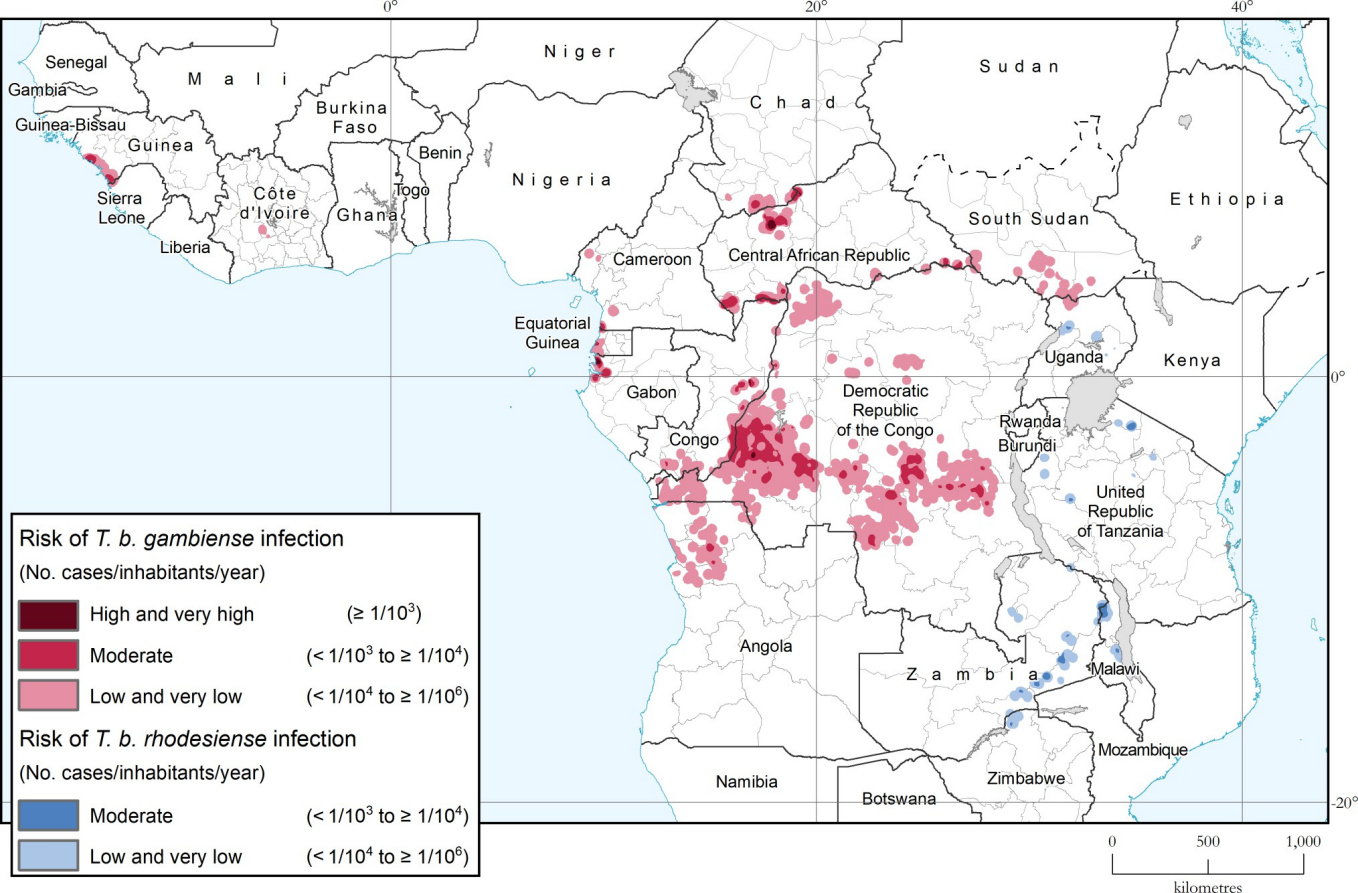
Areas at risk of HAT infection. Period 2016–2020. The base layers used in this map are the FAO Global Administrative Unit Layers (GAUL), Global Administrative Areas and FAO Inland water bodies in Africa.

In 2019 and 2020 the areas at very high, high or moderate risk of HAT continued to decline steadily, as already observed during the past decade (Fig 3). At the continental level, the areas reporting ≥ 1 case/10,000 inhabitants/year cover a surface of 120,000 km^2^ (period 2016–2020), corresponding to an 83% reduction from the 2000–2004 baseline and being only 7% short of the reduction target of 90%. It is worth noting that the reduction was comparatively larger for the higher risk category (≥ 1 case/1000 inhabitants/year), which shrunk by 99% between 2000–2004 and 2016–2020. Fig 4 shows the geographical distribution of areas at various levels of risk for the period 2016–2020. S2 File provides the country breakdown, S3 File the larger-scale subregional maps, and S4 File a video of the areas at risk from 2000–2004 to 2016–2020.

The population at risk of HAT is estimated to be a total of 55 million for the period 2016–2020; however, only 6% (i.e. 3 million people) are considered at moderate or higher risk, compared with 11% for the period 2014–2018 [7]. The country breakdown for the population at risk is provided in S5 File.

### Population at risk potentially covered by fixed health facilities with capacities for HAT diagnosis and treatment

#### Survey and mapping of fixed health facilities

**Fig 5:**
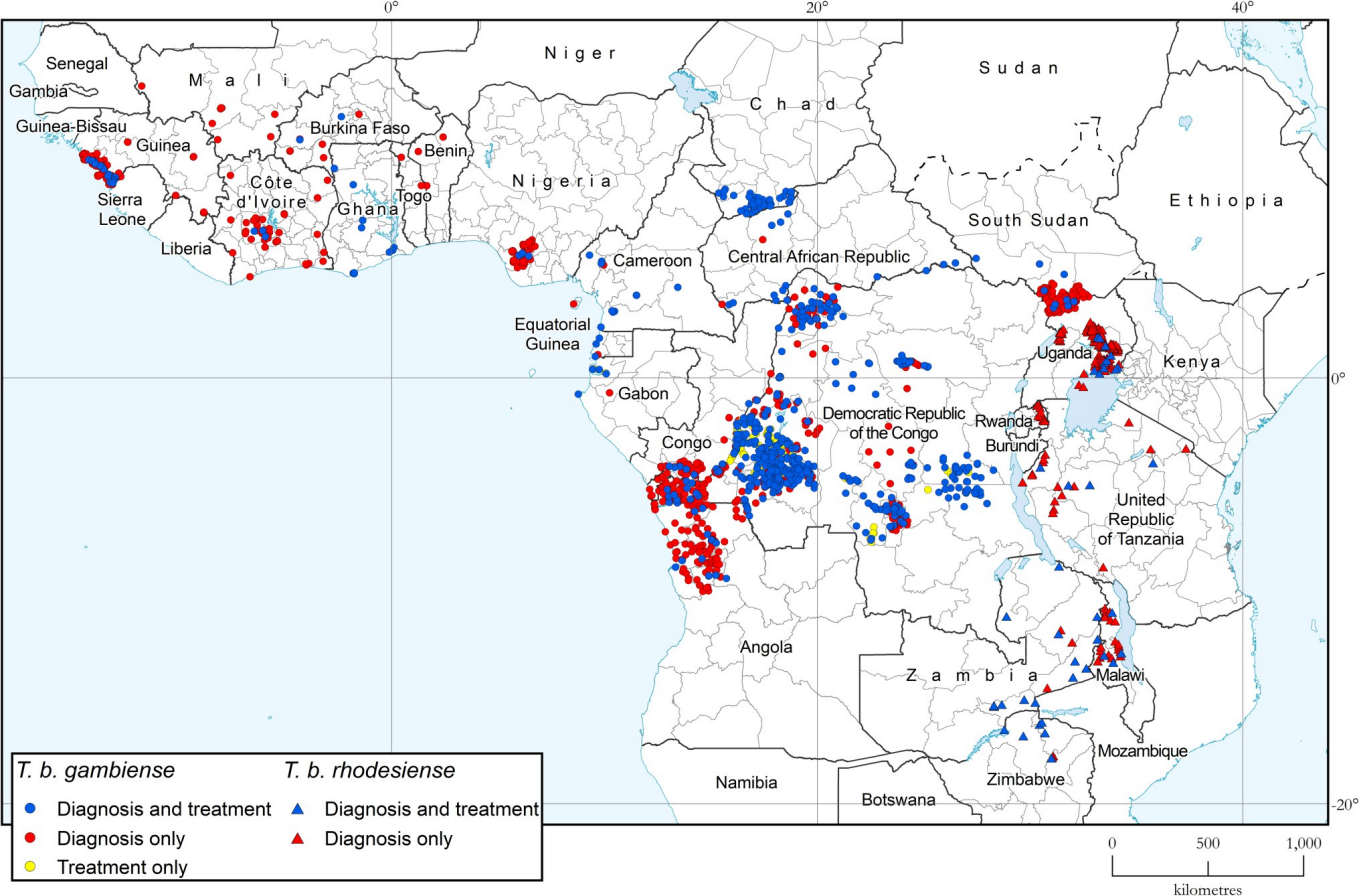
Geographical distribution of fixed health facilities offering diagnosis and treatment of gambiense and rhodesiense HAT. Data were collected by WHO from National Sleeping Sickness Control Programmes in June 2021. The base layers used in this map are the FAO Global Administrative Unit Layers (GAUL), Global Administrative Areas and FAO Inland water bodies in Africa.

Tables 3 and 4 show the fixed health facilities with capacity for diagnosis or treatment of gambiense and rhodesiense HAT respectively; their geographical distribution is shown in Fig 5. Data are based on the WHO survey that was completed in June 2021.

**Table 3:** Fixed health facilities for gambiense HAT: June 2021 survey. Differences from the July 2019 survey [7] in column ‘Δ’.

Country	Diagnosis						Treatment						TOTAL	Δ
	DxC	DxS	DxP	DxPh	Total	Δ	Tx1P	Tx2M	Tx2F	Tx2N	Total	Δ		
Angola	121	121	25	12	121	-9	12	5	0	10	12	-9	121	-9
Benin	3	3	0	0	3	0	0	0	0	0	0	0	3	0
Burkina Faso	8	8	2	2	8	0	2	2	2	0	2	0	8	0
Cameroon	13	10	9	6	14	0	11	0	7	8	11	0	14	0
Central African Republic	16	9	8	8	16	1	13	3	12	11	14	4	16	1
Chad	54	53	8	8	54	2	54	4	7	10	54	1	54	1
Congo	55	55	8	8	55	-13	7	0	0	7	7	4	55	-13
Côte d’Ivoire	40	40	3	3	40	11	3	2	0	3	3	1	40	11
Democratic Republic of the Congo	821	721	325	262	821	125	581	69	166	199	587	41	917	118
Equatorial Guinea	4	4	2	1	4	0	2	1	1	1	2	-2	4	0
Gabon	7	7	1	1	7	2	2	0	6	3	7	6	9	3
Ghana	10	10	0	0	10	0	10	0	0	0	10	0	10	0
Guinea	129	127	3	3	129	4	3	2	22	3	22	19	129	4
Mali	7	7	3	0	8	0	0	0	0	0	0	0	8	0
Nigeria	50	50	5	5	50	0	5	0	0	5	5	0	50	0
South Sudan	77	76	6	6	77	17	5	0	0	5	5	0	77	17
Togo	2	2	0	0	2	0	0	0	0	0	0	0	2	0
Uganda	53	53	12	4	53	5	4	4	0	4	4	0	53	5
**Total**	1,470	1,356	420	329	1,472	145	714	92	223	269	745	65	1,570	138
**Δ**	145	164	53	32	145		34	5	20	14	65		138	

DxC: clinical diagnosis; DxS: serological diagnosis; DxP: parasitological diagnosis; DxPh: disease staging. Tx1P: treatment of first-stage infection with pentamidine; Tx2M: treatment of second-stage infection with melarsoprol; Tx2F: treatment of second-stage infection with fexinidazole; Tx2N: treatment of second-stage infection with nifurtimox-eflornithine combination therapy (NECT).

**Table 4:** Fixed health facilities for rhodesiense HAT: June 2021 survey. Differences from the July 2019 survey [7] in column ‘Δ’.

Country	Diagnosis					Treatment				TOTAL	Δ
	DxC	DxP	DxPh	Total	Δ	Tx1S	Tx2M	Total	Δ		
Kenya	1	1	1	1	0	1	1	1	0	1	0
Malawi	24	15	4	24	3	4	4	4	0	24	3
Rwanda	12	12	4	12	0	0	0	0	0	12	0
Uganda	145	143	12	145	107	10	10	10	0	145	107
United Republic of Tanzania	20	5	3	20	3	4	4	4	0	20	3
Zambia	18	18	18	18	2	15	15	15	1	18	2
Zimbabwe	8	6	6	8	2	6	1	6	0	8	2
**Total**	228	200	48	228	117	40	35	40	1	228	117
**Δ**	117	111	2	117		1	1	1		117	

DxC: clinical diagnosis; DxP: parasitological diagnosis; DxPh: disease staging. Tx1S: treatment of first-stage infection with suramin; Tx2M: treatment of second-stage infection with melarsoprol.

For gambiense HAT, 1,570 fixed health facilities with capacity for diagnosis and/or treatment were reported, corresponding to a 9.6% increase from the 2019 survey [7]. Diagnosis is available in 1,472 facilities (+10.9%) and treatment in 745 (+9.6%). More than half of the facilities (58.4%) are in the Democratic Republic of the Congo. For rhodesiense HAT, 228 facilities (+105.4%) offer diagnosis in seven endemic countries (Kenya, Malawi, Rwanda, Uganda, United Republic of Tanzania, Zambia and Zimbabwe) and treatment is provided in 40 health facilities (+2.6%).

The increase in gambiense HAT facilities was driven by the Democratic Republic of the Congo, which added 125 for diagnosis and 41 for treatment, but relatively significant increases were also reported from South Sudan (+17) and Côte d’Ivoire (+11). Only two countries reported a reduction: Congo (-13) and Angola (-9).

For rhodesiense HAT, the doubling of facilities offering diagnosis can be credited to one single country–Uganda–which added 107, related to increased capacities in the context of a clinical trial. Diagnostic facilities remained largely unchanged in other countries, as did treatment facilities overall, including in Uganda.

No specific health facility with capacity to diagnose and treat HAT is reported from Botswana, Burundi, Eswatini, Ethiopia, the Gambia, Guinea-Bissau, Liberia, Mozambique, Namibia, Niger, Senegal and Sierra Leone.

#### Population at risk potentially covered by fixed health facilities

The potential coverage of the population at risk of HAT by fixed health facilities is summarized in S6 File. For gambiense HAT, approximately 33, 43 and 48 million people at risk are respectively within one, three and five hours’ travel from a facility offering diagnosis. These values correspond to 62%, 82% and 92% of the at-risk population in the period 2016–2020, marking a very slight improvement over the 2014–2018 values when 60%, 82% and 91% of the at-risk population was within one, three and five hours’ travel [7]. For treatment, the population at risk within the same travel time thresholds is 27 million (52% of the at-risk population), 41 million (79%) and 47 million (90%), again only marginally better than in the 2014–2018 period.

For rhodesiense HAT, the proportion of the at-risk population potentially covered by fixed health facilities is lower than that for gambiense HAT. Specifically, 30%, 66% and 83% of the rhodesiense HAT at-risk population is within one, three and five hours’ travel of a facility offering diagnosis. For treatment, the corresponding values are 21%, 54% and 75%. These values are lower than those of the 2014–2018 period (i.e. 35%, 67% and 84% for diagnosis and 27%, 58% and 75% for treatment [7]).

### Vector control

In the period 2019–2020, vector control activities targeted at gambiense HAT have been implemented by the NSSCP and research institutions in selected endemic areas of Cameroon, Chad [32,33], Côte d’Ivoire [34], Democratic Republic of the Congo [35], Guinea [36] and Uganda [37]. Vector control activities are also carried out by the NSSCP in Angola. Vector control decreased tsetse densities in the targeted areas and contributed to curbing HAT transmission.

Tsetse control activities are also carried out in a number of countries in which rhodesiense HAT is endemic, including Kenya [38], Malawi [39], United Republic of Tanzania [40,41], Uganda [42], Zambia [43] and Zimbabwe [44], where interventions against the tsetse vector are primarily driven by the need to control animal trypanosomiasis; interventions are normally implemented by veterinary services and livestock keepers. However, the benefits to One Health of tsetse control are well recognized both nationally and internationally [45], and they are explicitly pursued in many areas [46].

### HAT elimination as a public health problem at country level

By the end of 2020, two countries had received official validation of HAT elimination as a public health problem: Togo, in August 2020, and Côte d’Ivoire, in December 2020. By the same date, three additional countries had submitted their dossier to WHO for evaluation and validation and a further three were preparing their dossier.

## Discussion

The epidemiological patterns of HAT described in this paper for the period 2019–2020 follow the trends observed for over a decade. In particular, the main global target for the elimination of HAT as a public health problem by 2020 (i.e. < 2,000 cases reported/year) had already been attained in 2017. This achievement was sustained and improved subsequently, with 992 cases reported in 2019 and 663 in 2020. For the second global indicator, the area at moderate or higher risk of HAT has been shrinking constantly for more than a decade. However, in 2016–2020, with an 83% reduction compared with the 2000–2004 baseline, the target of 90% has not been fully reached yet. It is worth noting that the reduction was 99% for the high and very high risk category, and 73% for the moderate risk category, meaning that the remaining areas at risk are much more skewed towards lower risk categories. If past trends are sustained, the second global target for HAT elimination as a public health problem could be fully achieved by 2022.

For gambiense HAT, which represents 97% of the total burden of HAT reported in the period 2001–2020, it is evident that its trends follow the pattern already discussed for HAT in general. Furthermore, it is worth noting that our current knowledge of the occurrence and geographical distribution of this form of the disease is more accurate and reliable now than it was a decade ago. This is because, over the years, both active screening and passive surveillance have been reinforced in the majority of endemic countries, thus strengthening the notion that the reported trends correspond to a real, strong reduction of HAT transmission in the field. Against this backdrop at the global level, the official process of validation of HAT elimination as a public health problem at the national level has started, with a number of countries already engaged.

Notwithstanding this positive general picture, the epidemiological situation of gambiense HAT varies between countries, and control and surveillance activities remain weak in some of them. Particularly noteworthy are the challenges to HAT control in the Central African Republic, South Sudan and in some areas of the Democratic Republic of the Congo, where weak health systems and insecurity hamper access to some endemic areas and render the reported epidemiological data less reliable. Our knowledge of the epidemiological situation is also limited in Nigeria because of shortcomings of HAT surveillance and control. Also, in the Gambia, Guinea-Bissau, Liberia [47], Niger [48], Senegal and Sierra Leone, an operational surveillance system for HAT is lacking because sleeping sickness, once present in these countries, is believed to be very rare today or absent altogether, most likely because of a combination of environmental, social and epidemiological changes.

For rhodesiense HAT, reported cases are sporadic but outbreaks are an ever-present risk. This was demonstrated between 2019 and the beginning of 2020, when cases surged in Malawi around the Vwaza Marsh Game Reserve and Nkhotakota Wildlife Reserve Park, as well as in some areas of Zambia. This spike in transmission appears to have been caused by changes in the contact patterns between people, tsetse and other animals, which in turn were triggered by climate variations, particularly extreme drought [49].

The level of under detection for rhodesiense HAT remains of concern. In the past few years the problem has been exacerbated by the extended use of rapid tests to diagnose malaria, and the corresponding decline in the use of blood smears [50]. In response, specific training sessions were undertaken to reinforce diagnostic capacities in some endemic countries (e.g. Malawi, Uganda, Zambia and Zimbabwe).

When interpreting recent data on HAT cases, it is impossible to overlook the impact of the COVID-19 pandemic in 2020 and the disruption caused to health services worldwide. Some HAT control activities that carry a higher risk of transmission of the SARS-CoV-2 virus were cancelled (e.g. active screening campaigns) [51]. Furthermore, health services redirected large shares of resources to fighting COVID-19, thereby reducing that for other routine services. This explains the reduction in the number of people screened for HAT, the higher-than-expected reduction in the number of HAT reported cases, and the likely higher level of under-detection. To tackle these problems, mitigation measures were recommended to reduce the impact of the pandemic on health services providing care for NTDs [52].

In 2020, Togo was the first country to submit its dossier to WHO to validate the elimination of HAT as a public health problem at the national level. Following the procedures established by WHO, the dossier was examined by an independent panel of experts, which deemed the presented data sufficient to validate the claim. A similar exercise was subsequently undertaken by a number of other countries and is already completed by Côte d’Ivoire. An important element in the process of validation is the planning of activities to sustain the surveillance system, which should be able to detect cases and trigger an adequate response in case of re-emergence or reintroduction of the disease.

## Conclusions

The work and commitment of NSSCPs, the sustained public–private partnership and the coordinating and enabling role played by WHO have been the cornerstones of the achievements presented in this paper. In particular, the donation of the necessary medicines by manufacturers was essential to ensure access to treatment for all diagnosed patients. The contributions of academic institutions and philanthropic organizations in improving diagnostic and treatment tools and enhancing our epidemiological understating of HAT were also important in the fight against the disease.

At this juncture–when the goal of HAT elimination as a public health problem is within grasp–it is crucial to remember that it is just a stepping stone towards the more ambitious goal of elimination of gambiense HAT transmission. Through extensive consultations with endemic countries and partners, this goal was included in the new WHO road map for NTDs [53], and it was officially endorsed in a virtual session of the 73rd World Health Assembly. The new goal of elimination is set to be achieved for 2030, which will be particularly demanding as it requires sustaining and adapting interventions in the context of low endemicity. The main global indicator of verifying the elimination of gambiense HAT was defined as zero reported cases, which must be supported by an effective surveillance system. Additionally, the process of validating the elimination of HAT as public health problem at the national level must continue, beyond which countries must move towards verification of the sustainable elimination of transmission [53].

An important epidemiological challenge in the elimination of gambiense HAT is the role that cryptic reservoirs such as asymptomatic human carriers and non-human animals could play in maintaining or rekindling transmission [8]. On the road towards the elimination of transmission, other factors also become critical. First, ownership and leadership of control and surveillance activities by national health programmes must be sustained and reinforced. Also, integration of HAT activities in the health system is increasingly needed. New, easier-to-use and more effective tools currently in the pipeline are expected to help the integration process. These new tools include a single-dose oral treatment for both stages of the disease (acoziborole [54,55]), performant and easier-to-produce second generation rapid diagnostic tests [56,57], and high throughput referral tests performed in dry blood spots not requiring sophisticated transport conditions (ELISA [58] and other molecular tests [59,60]). The adaptation of control strategies to the evolving patterns of disease transmission was one of the keys to success in past years, and it should continue in the future. In this context, Geographic Information Systems and the systematic mapping of epidemiological and control data are becoming ever more critical for planning, targeting and monitoring interventions [3,6,7,22–24,27].

The COVID-19 pandemic has given a stark reminder of how the process of HAT elimination can be threatened by unexpected challenges. At the same time, it has shown the resilience of national programmes, and their ability to react, adapt and maintain a minimum level of activities even in difficult times.

Rhodesiense HAT remains targeted for elimination as a public health problem in the new NTD road map for 2021–2030. As a relatively rare zoonosis, further advances towards elimination are not easy to make, and they will require control of infections in non-human animals in a One Health framework [45]. Unfortunately, especially for the wildlife reservoir, control options are currently limited and rely mainly on vector control. The past biennium reminded us that unexpected outbreaks of rhodesiense HAT, such as the one in Malawi, are always a possibility, and that effective surveillance is crucial for early detection, early response and, ultimately, for successful control.

For both gambiense and rhodesiense HAT the continued commitment of resource partners will be essential to sustain the gains made thus far and to advance towards the 2030 goals. It is very encouraging that the main donors have signalled interest in continuing their engagement in HAT elimination. Notably, the pharmaceutical companies Sanofi and Bayer have committed to ensuring access to treatment for the next five years, including for the new medicines that will be released during this period. Conversely, access to screening and diagnostic tools is not guaranteed, and even their production is not secured. This is a cause for concern that warrants the urgent attention of donors and resource partners, as it introduces deep uncertainties in the planning of the elimination process.

As we stand at the brink of attaining the goal of HAT elimination as a public health problem, the main lesson that can be drawn from this success is that ambitious goals can be achieved even with imperfect control tools, as long as national programmes are fully committed and the support of international stakeholders is sustained and coordinated. Another lesson is that gathering accurate epidemiological data is vital to design adapted intervention strategies and to monitor impacts. These lessons should guide us as we embark on the next challenging endeavours.

## Supporting information

S1 File Geographical distribution of human African trypanosomiasis, subregional maps. Period 2019–2020. The base layers used in the maps are the FAO Global Administrative Unit Layers (GAUL), Global Administrative Areas, Shuttle Radar Topography Mission (SRTM), FAO Inland water bodies in Africa, FAO Rivers of Africa and Vector Map Level 0 (VMap0).
(PDF)

S2 File Area at risk of gambiense and rhodesiense HAT. Period 2016–2020 (by country).
(DOCX)

S3 File Areas at risk of HAT infection, subregional maps. Period 2016–2020. The base layers used in the maps are the FAO Global Administrative Unit Layers (GAUL), Global Administrative Areas and FAO Inland water bodies in Africa.
(PDF)

S4 File Slide show of the areas at risk of HAT from 2000–2004 to 2016–2020. The base layers used in the maps are the FAO Global Administrative Unit Layers (GAUL) and FAO Inland water bodies in Africa.
(PPSX)

S5 File Population at risk of gambiense and rhodesiense HAT. Period 2016–2020 (by country).
(DOCX)

S6 File People at risk of HAT who are potentially covered by facilities with diagnostic and treatment capabilities for HAT. (DOCX)
