# Supplementary material for: The elimination of human African trypanosomiasis: Achievements in relation to WHO road map targets for 2020
Source: PLoS Negl Trop Dis. 2022 Jan 18;16(1):e0010047. doi: 10.1371/journal.pntd.0010047 (PMC8765662; doi:10.1371/journal.pntd.0010047)

Gambiense HAT cases in Western Africa. Period 2019–2020.

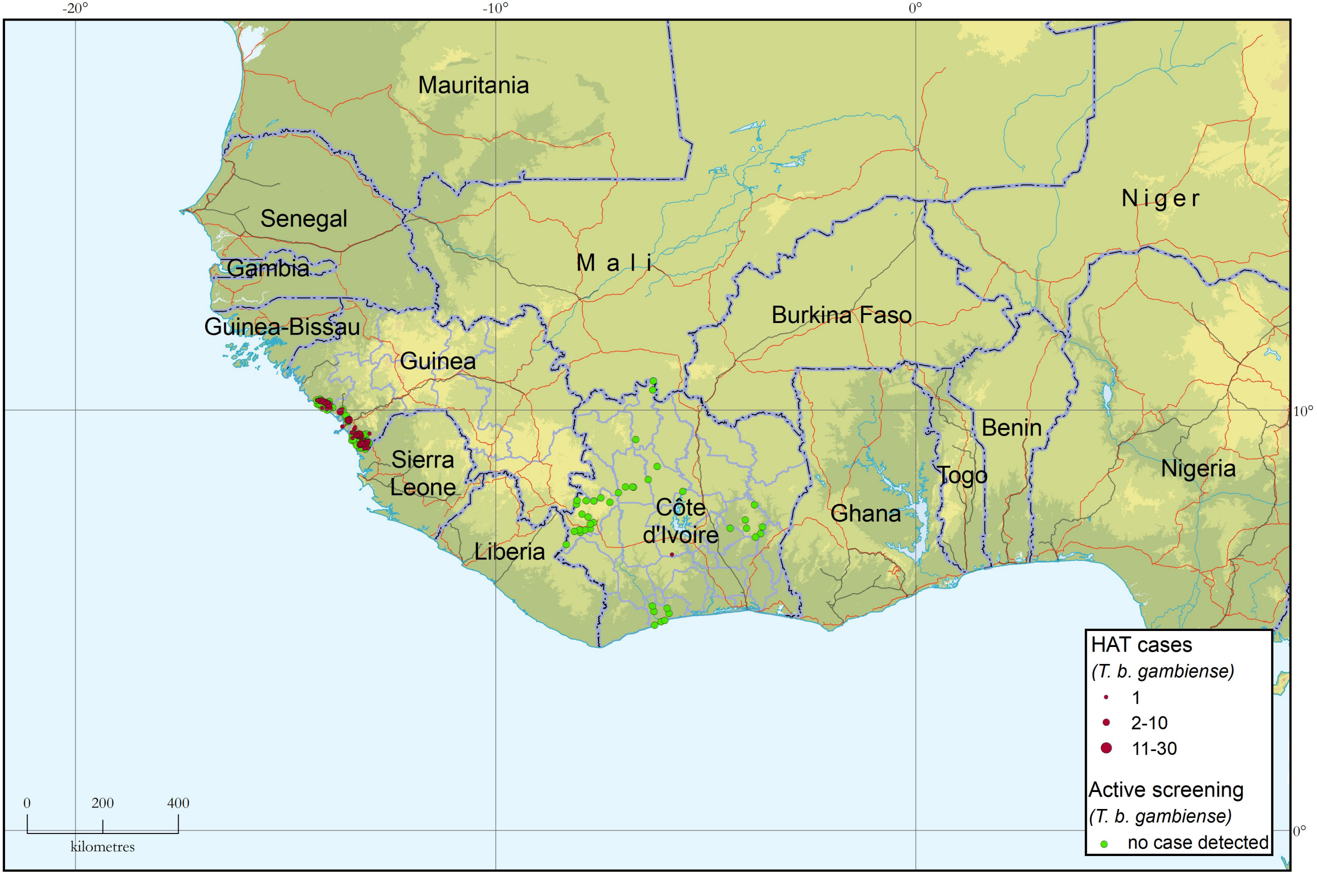

Gambiense HAT cases in Central Africa. Period 2019–2020.

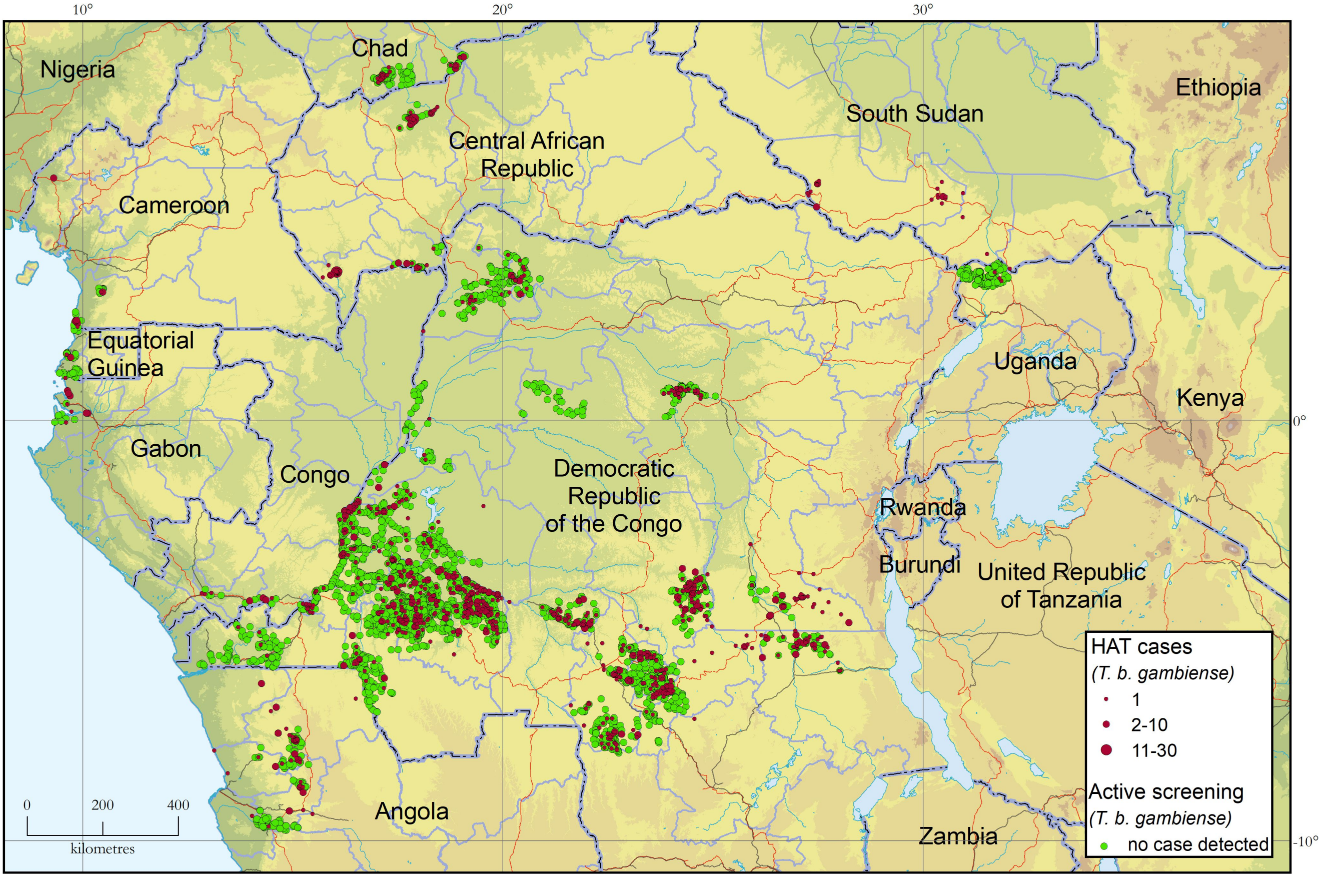

Rhodesiense HAT cases in Estern and Southern Africa. Period 2019–2020.

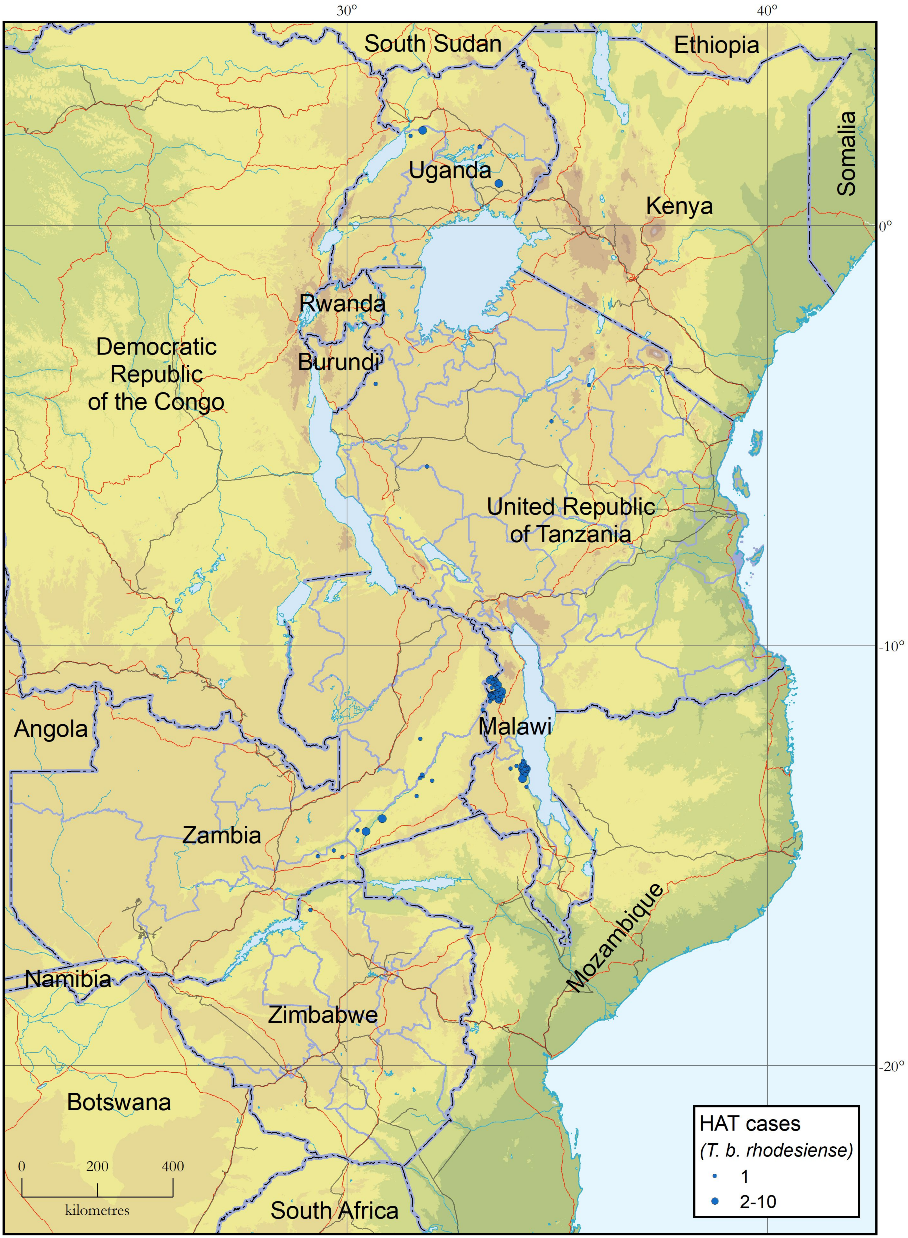

Supplement: S1 File — Period 2019–2020. The base layers used in the maps are the FAO Global Administrative Unit Layers (GAUL), Global Administrative Areas, Shuttle Radar Topography Mission (SRTM), FAO Inland water bodies in Africa, FAO Rivers of Africa and Vector Map Level 0 (VMap0). (PDF) [file pntd.0010047.s001.pdf]
