# Supplementary material for: The elimination of human African trypanosomiasis: Achievements in relation to WHO road map targets for 2020
Source: PLoS Negl Trop Dis. 2022 Jan 18;16(1):e0010047. doi: 10.1371/journal.pntd.0010047 (PMC8765662; doi:10.1371/journal.pntd.0010047)
Supplement: S2 File — Period 2016–2020 (by country). (DOCX) [file pntd.0010047.s002.docx]

# Area at risk of gambiense and rhodesiense HAT

Table 1 Areas at risk of *T. b. gambiense* infection (km^2^). Period 2016–2020.

| **Country** | **Total country area*** | **Area at risk**  **2016-2020** | | | | |
| --- | --- | --- | --- | --- | --- | --- |
|  |  | **Very High**  **and High** | **Moderate** | **Low and**  **Very Low** | **Total**  **at risk** | **% of total**  **country**  **area** |
| Angola | 1,253,770 | 0 | 1,647 | 64,078 | 65,725 | 5.2 |
| Burkina Faso | 274,470 | 0 | 0 | 0 | 0 | 0 |
| Cameroon | 466,396 | 0 | 1,132 | 6,871 | 8,003 | 1.7 |
| Central African Republic | 624,398 | 1,658 | 14,399 | 32,802 | 48,859 | 7.8 |
| Chad | 1,272,490 | 0 | 2,542 | 11,594 | 14,136 | 1.1 |
| Congo | 338,522 | 224 | 8,772 | 33,868 | 42,864 | 12.7 |
| Côte d'Ivoire | 321,363 | 0 | 0 | 2,264 | 2,264 | 0.7 |
| Democratic Republic of the Congo | 2,304,080 | 571 | 72,504 | 433,947 | 507,022 | 22.0 |
| Equatorial Guinea | 27,019 | 0 | 698 | 5,078 | 5,776 | 21.4 |
| Gabon | 265,978 | 694 | 2,742 | 4,389 | 7,824 | 2.9 |
| Guinea | 246,094 | 114 | 3,169 | 7,165 | 10,449 | 4.2 |
| Sierra Leone | 72,777 | 0 | 20 | 1,197 | 1,217 | 1.7 |
| South Sudan | 633,356 | 0 | 0 | 34,835 | 34,835 | 5.5 |
| Uganda | 205,540 | 0 | 0 | 4,311 | 4,311 | 2.1 |
| Other Endemic Countries** | 4,097,446 | 0 | 0 | 0 | 0 | 0 |
| Total | 12,403,699 | 3,261 | 107,623 | 642,401 | 753,286 | 6.1 |

* Land area. The area of surface water bodies as depicted in the Shuttle Radar Topography Mission—River-Surface Water Bodies dataset is not included.

** Countries at marginal risk: Benin, Gambia, Ghana, Guinea-Bissau, Liberia, Mali, Niger, Nigeria, Senegal and Togo.

Table 2 Areas at risk of *T. b. rhodesiense* infection (km^2^). Period 2016–2020.

| **Country** | **Total country area*** | **Area at risk**  **2016-2020** | | | | |
| --- | --- | --- | --- | --- | --- | --- |
|  |  | **Very High**  **and High** | **Moderate** | **Low and**  **Very Low** | **Total**  **at risk** | **% of total**  **country**  **area** |
| Malawi | 94,758 | 0 | 2,465 | 6,527 | 8,992 | 9.5 |
| United Republic of Tanzania | 886,278 | 0 | 1,746 | 10,773 | 12,519 | 1.4 |
| Uganda | 205,540 | 0 | 317 | 6,814 | 7,131 | 3.5 |
| Zambia | 742,479 | 0 | 4,650 | 29,874 | 34,525 | 4.6 |
| Zimbabwe | 388,414 | 0 | 224 | 5,566 | 5,791 | 1.5 |
| Other Endemic Countries** | 3,947,508 | 0 | 0 | 0 | 0 | 0 |
| Total | 6,264,977 | 0 | 9,403 | 59,554 | 68,957 | 1.1 |

* Land area. The area of surface water bodies as depicted in the Shuttle Radar Topography Mission—River-Surface Water Bodies dataset is not included.

** Countries at marginal risk: Botswana, Burundi, Eswatini, Ethiopia, Kenya, Mozambique, Namibia and Rwanda.
