# Supplementary material for: The elimination of human African trypanosomiasis: Achievements in relation to WHO road map targets for 2020
Source: PLoS Negl Trop Dis. 2022 Jan 18;16(1):e0010047. doi: 10.1371/journal.pntd.0010047 (PMC8765662; doi:10.1371/journal.pntd.0010047)

Areas at risk of gambiense HAT infection in Western Africa. Period 2016–2020.

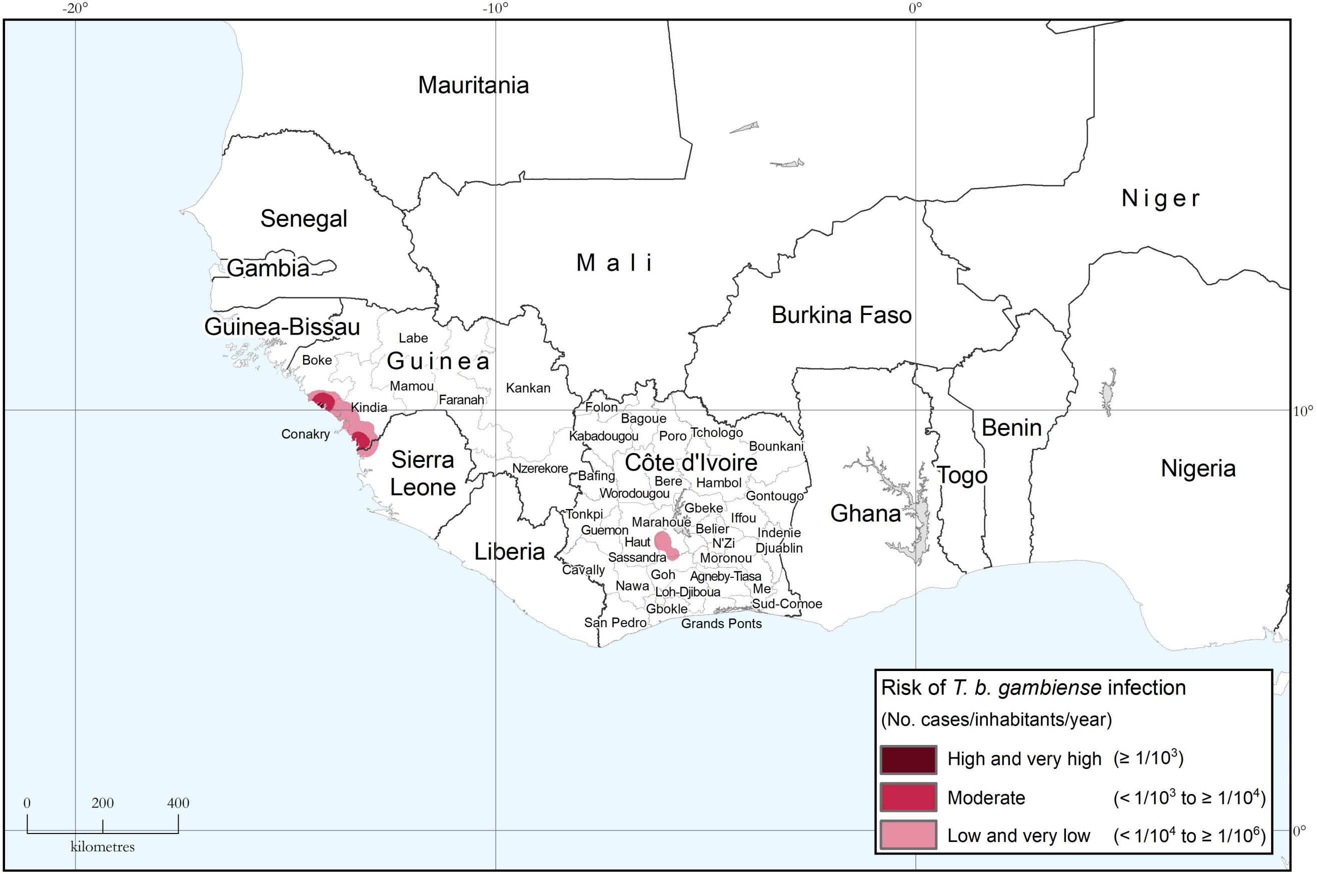

Areas at risk of gambiense HAT infection in Central Africa. Period 2016–2020.

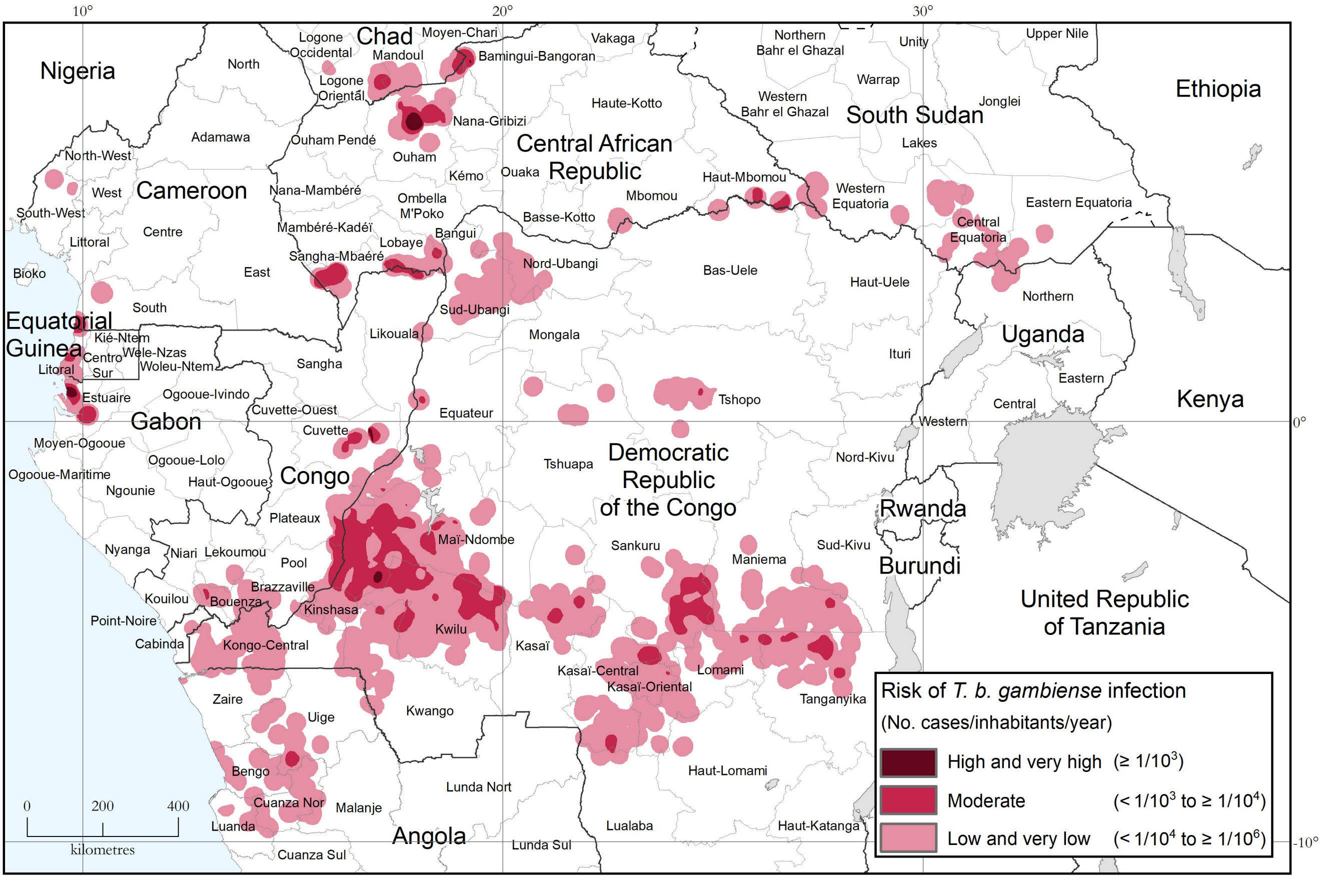

Areas at risk of rhodesiense HAT infection in Eastern and Southern Africa. Period 2016–2020.

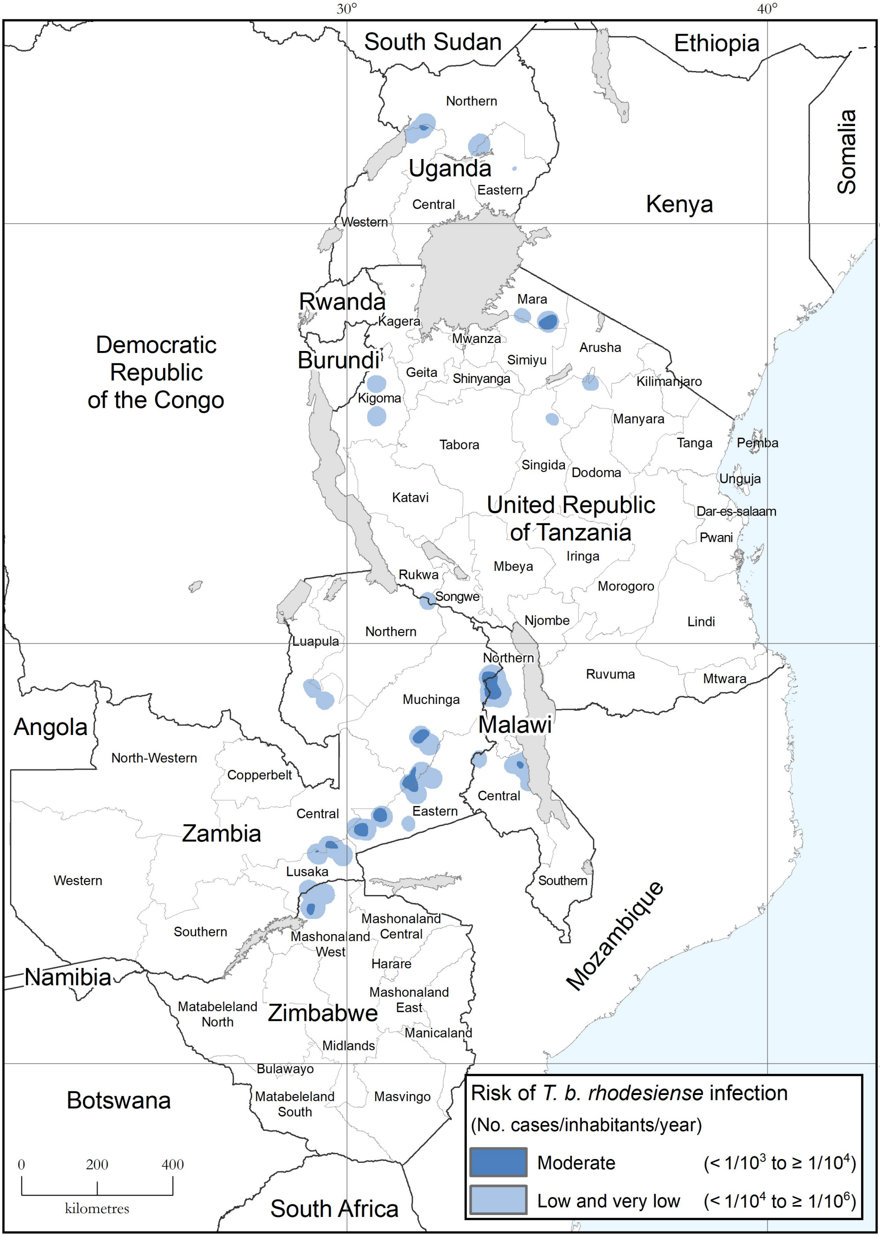

Supplement: S3 File — Period 2016–2020. The base layers used in the maps are the FAO Global Administrative Unit Layers (GAUL), Global Administrative Areas and FAO Inland water bodies in Africa. (PDF) [file pntd.0010047.s003.pdf]
