# Supplementary material for: The elimination of human African trypanosomiasis: Achievements in relation to WHO road map targets for 2020
Source: PLoS Negl Trop Dis. 2022 Jan 18;16(1):e0010047. doi: 10.1371/journal.pntd.0010047 (PMC8765662; doi:10.1371/journal.pntd.0010047)

## Slide 1
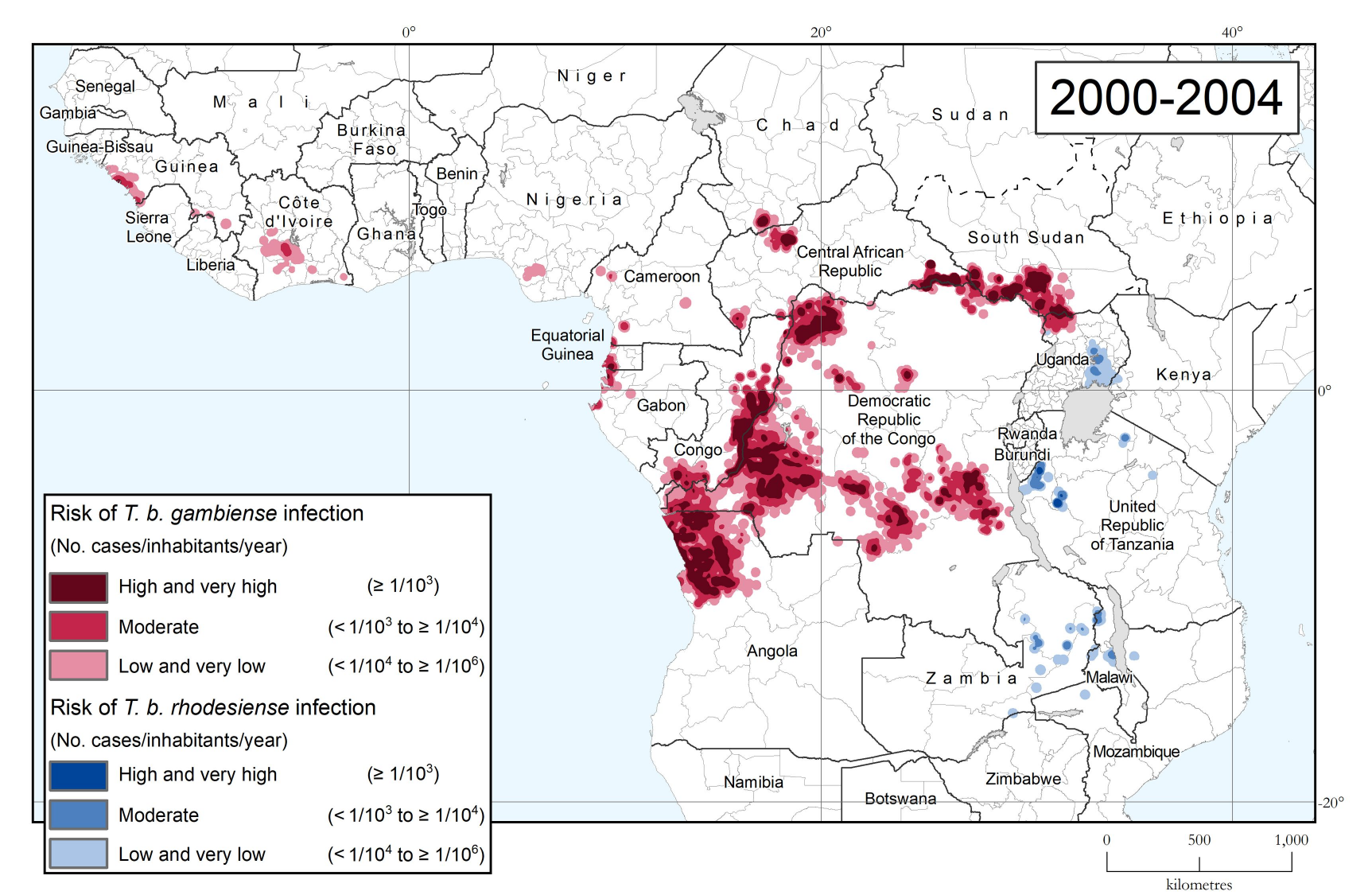

## Slide 2
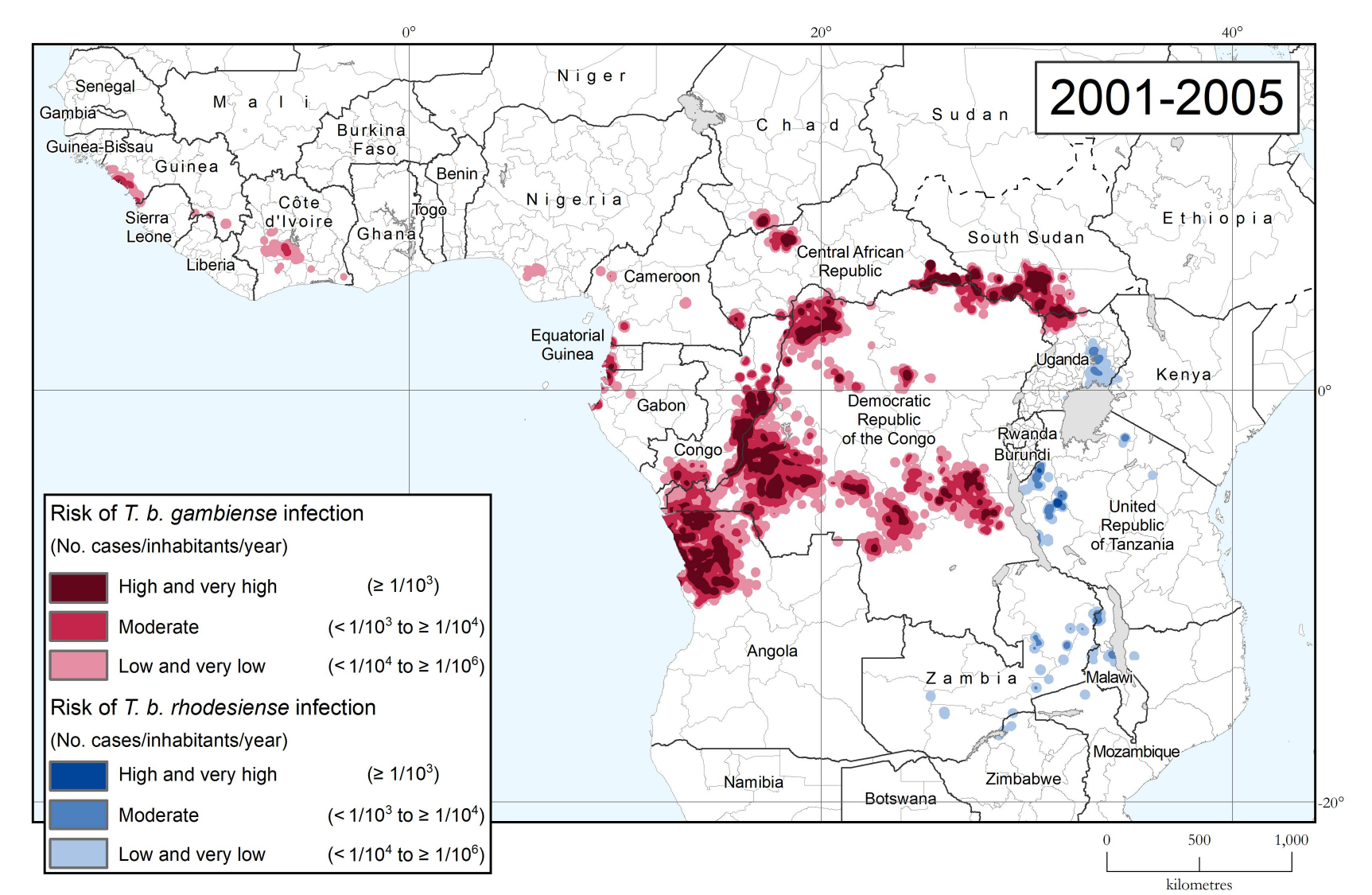

## Slide 3
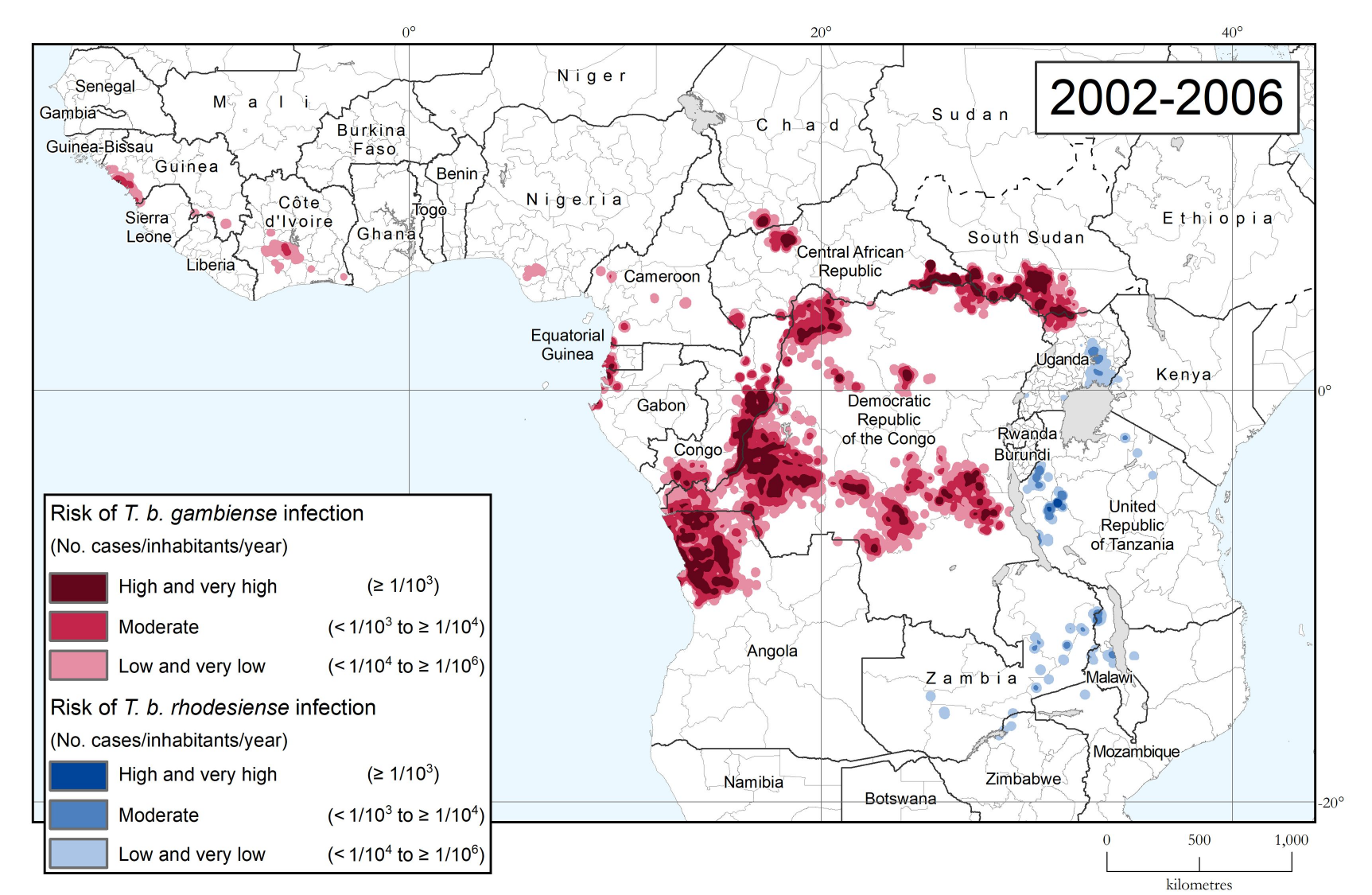

## Slide 4
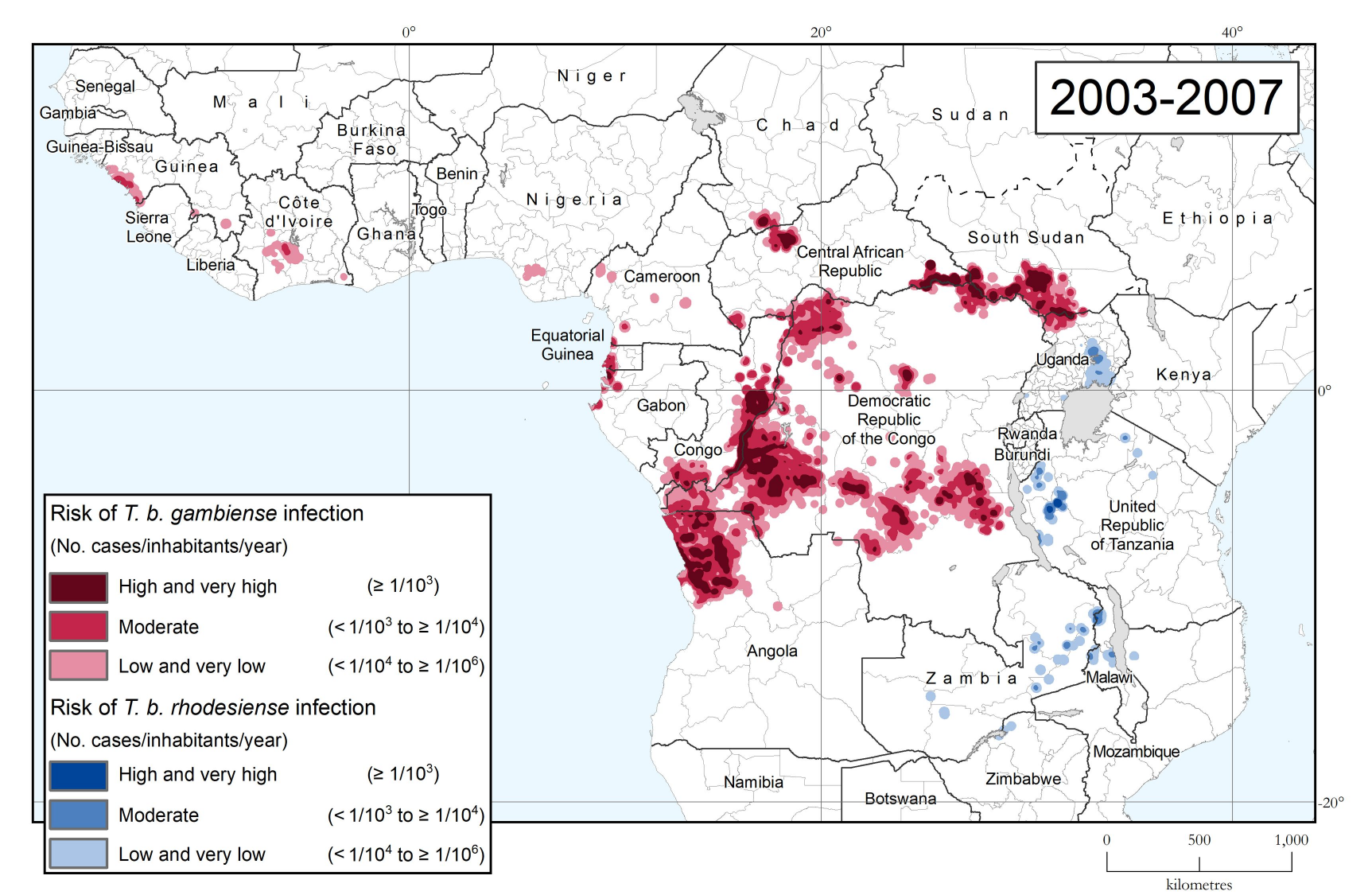

## Slide 5
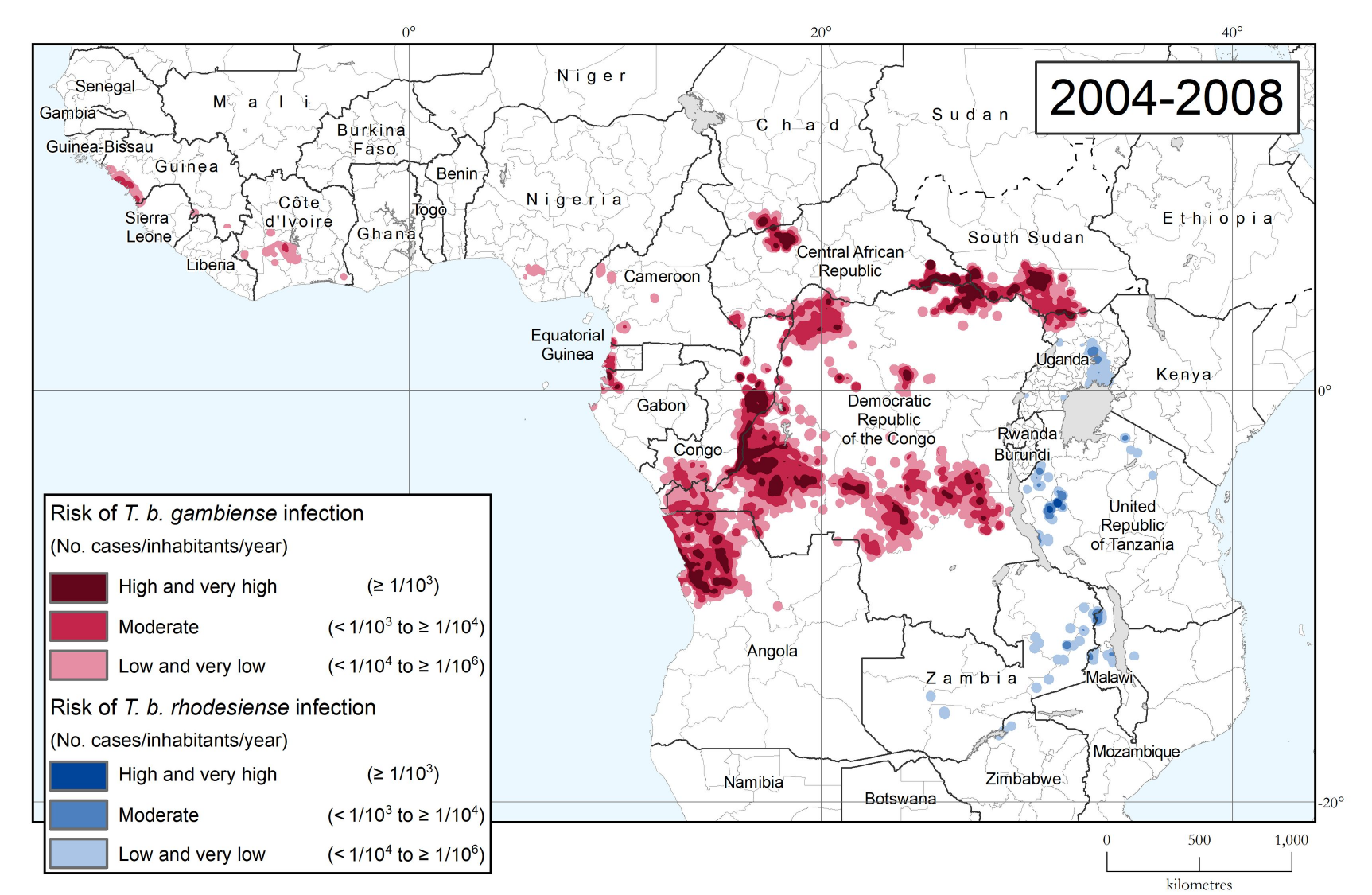

## Slide 6
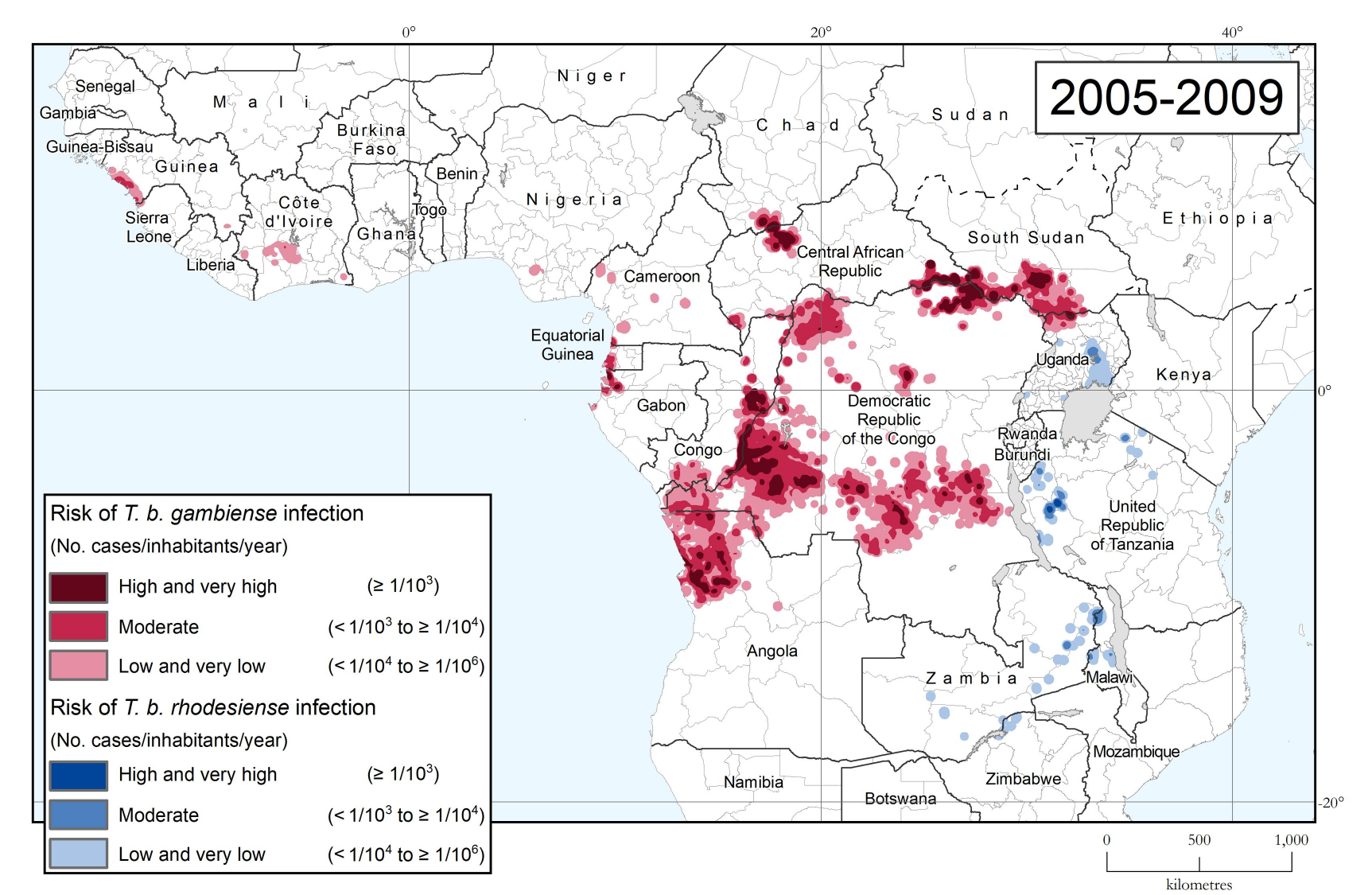

## Slide 7
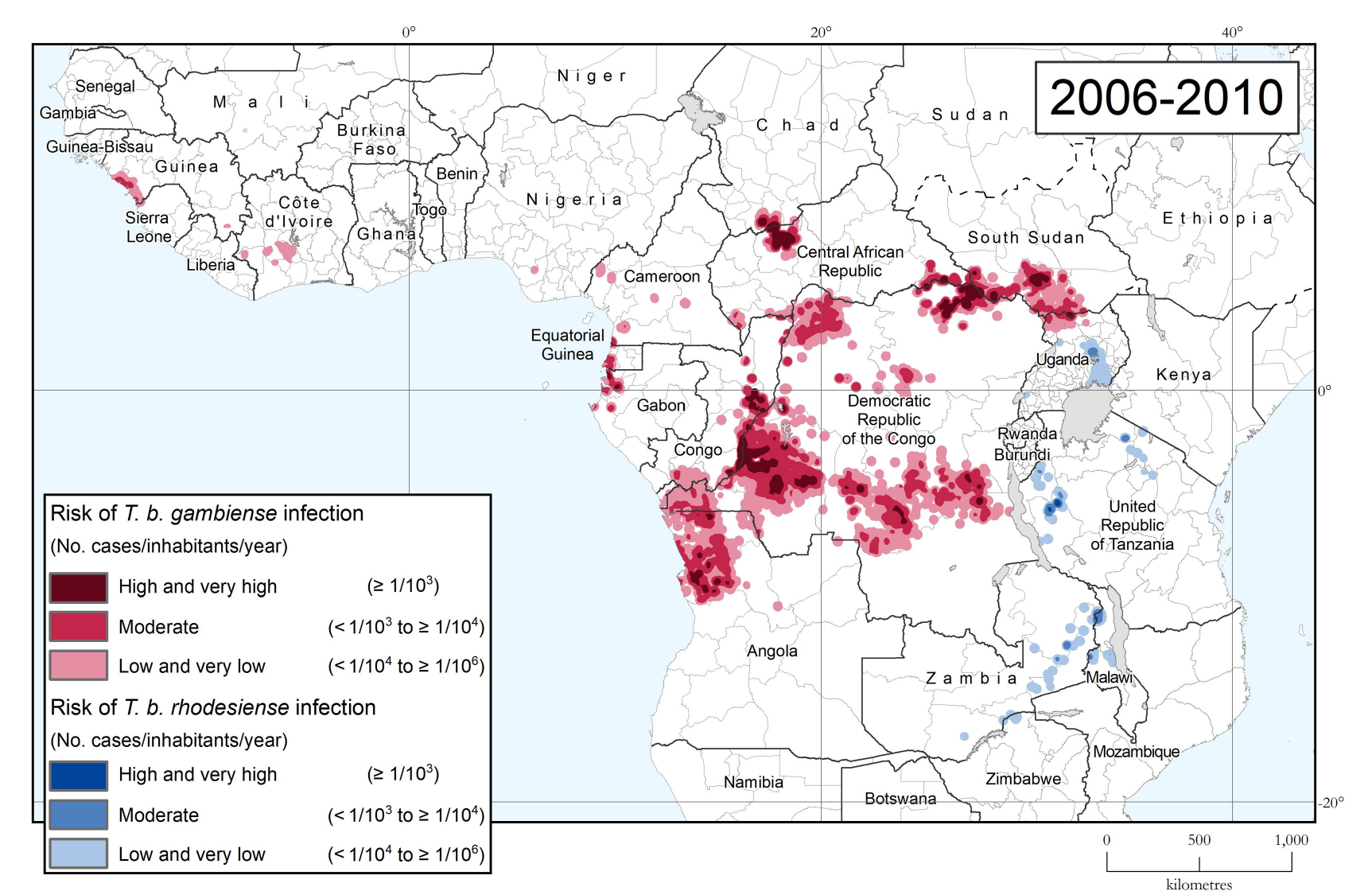

## Slide 8
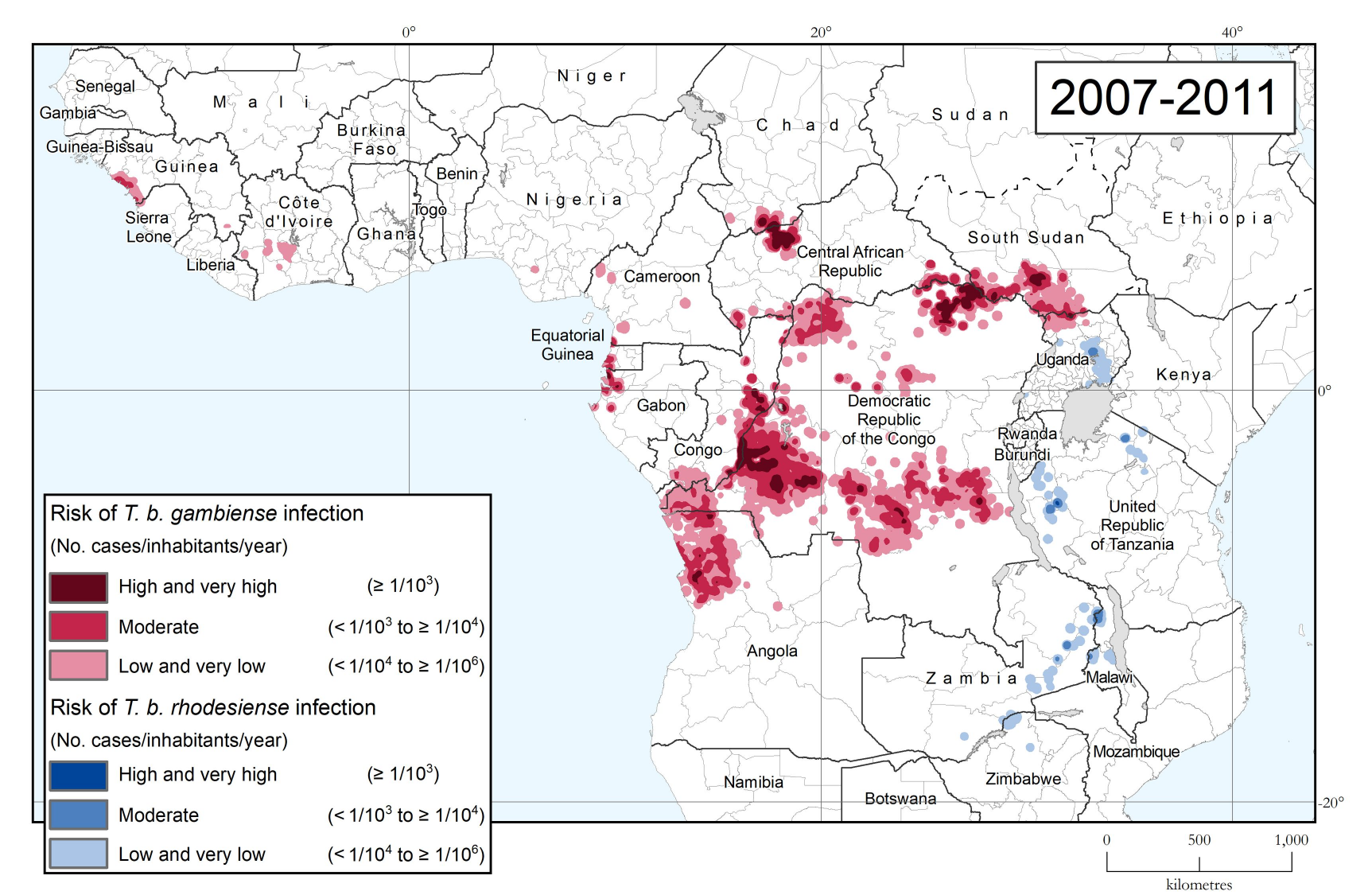

## Slide 9
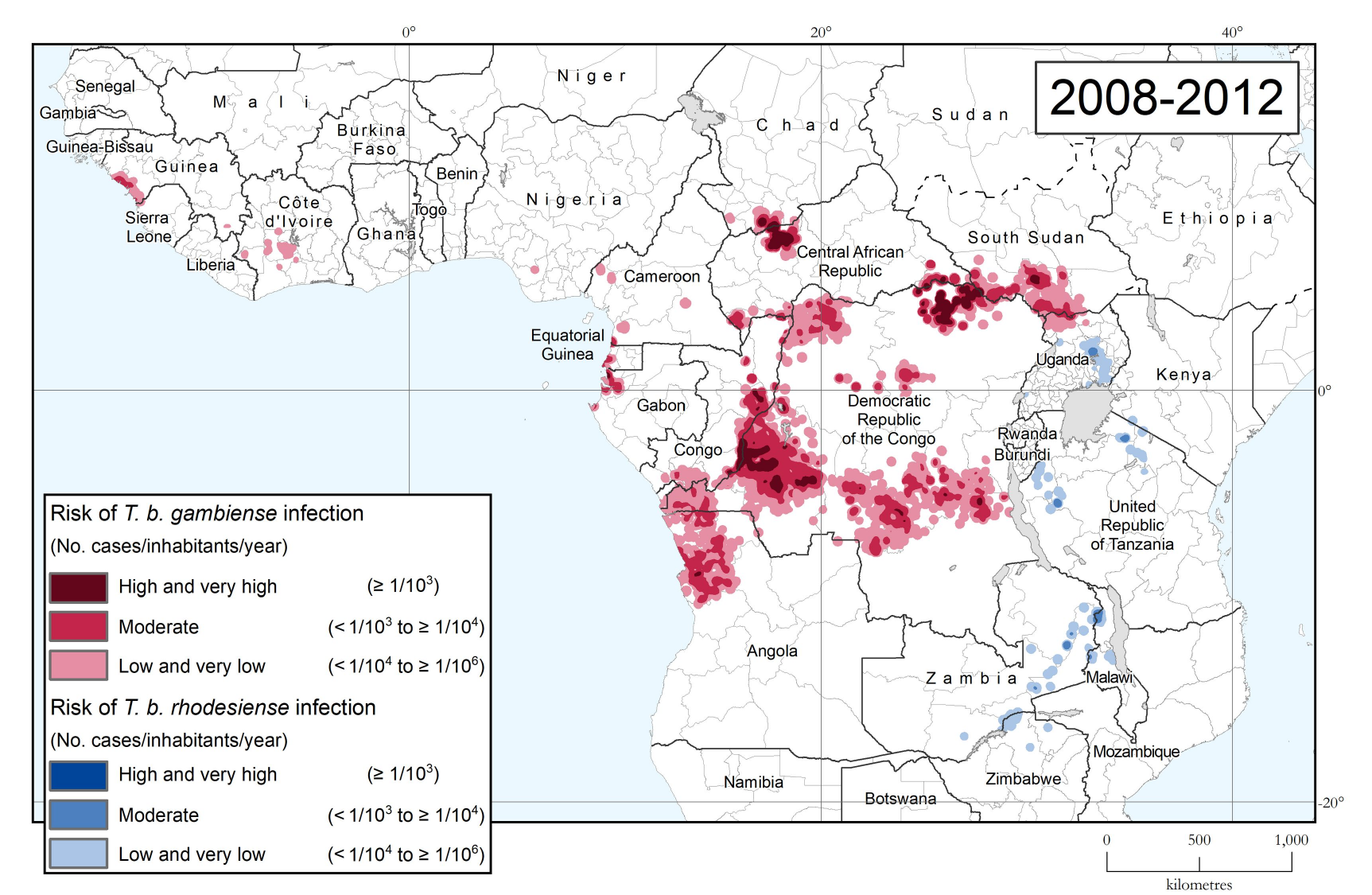

## Slide 10
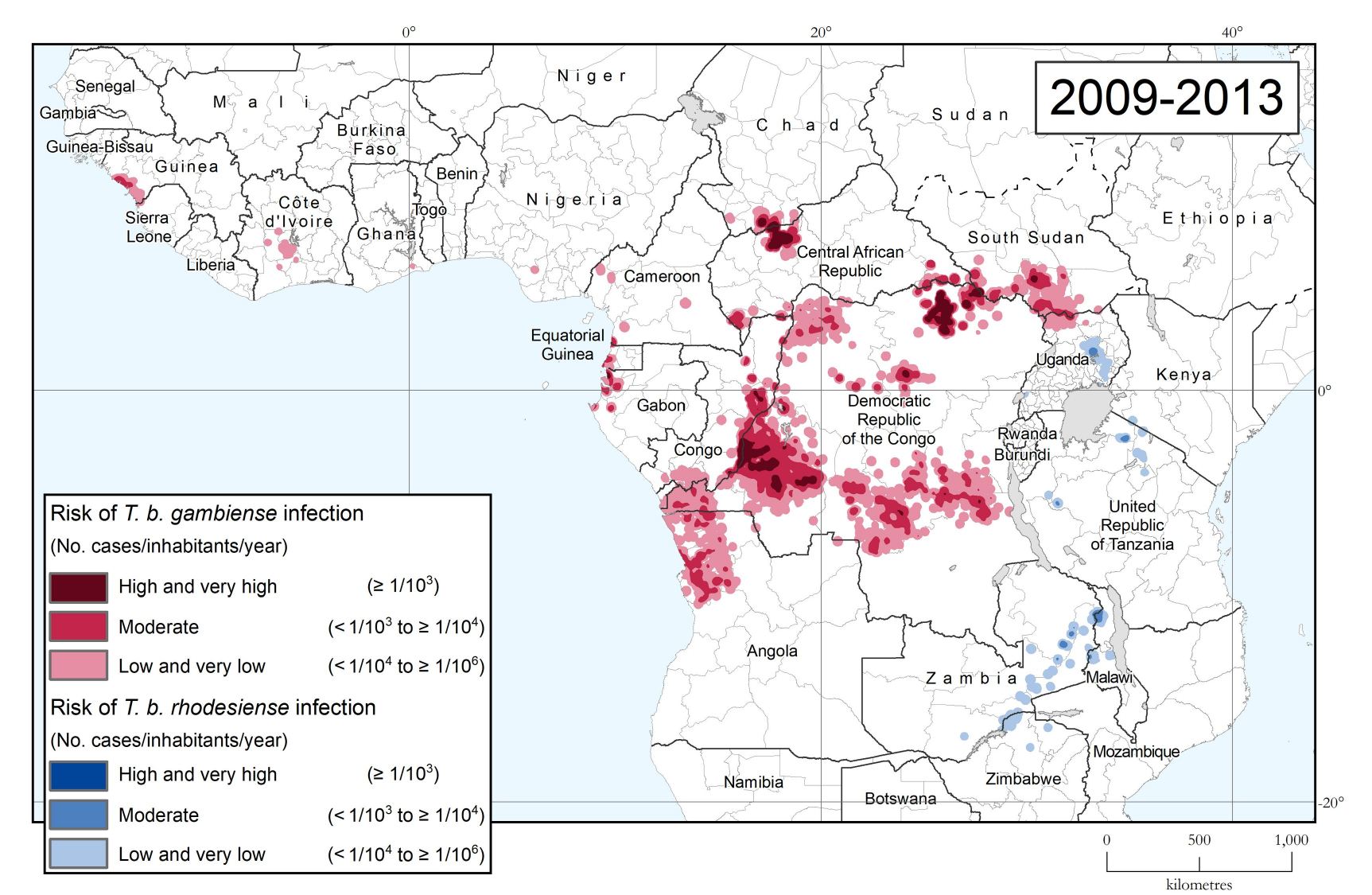

## Slide 11
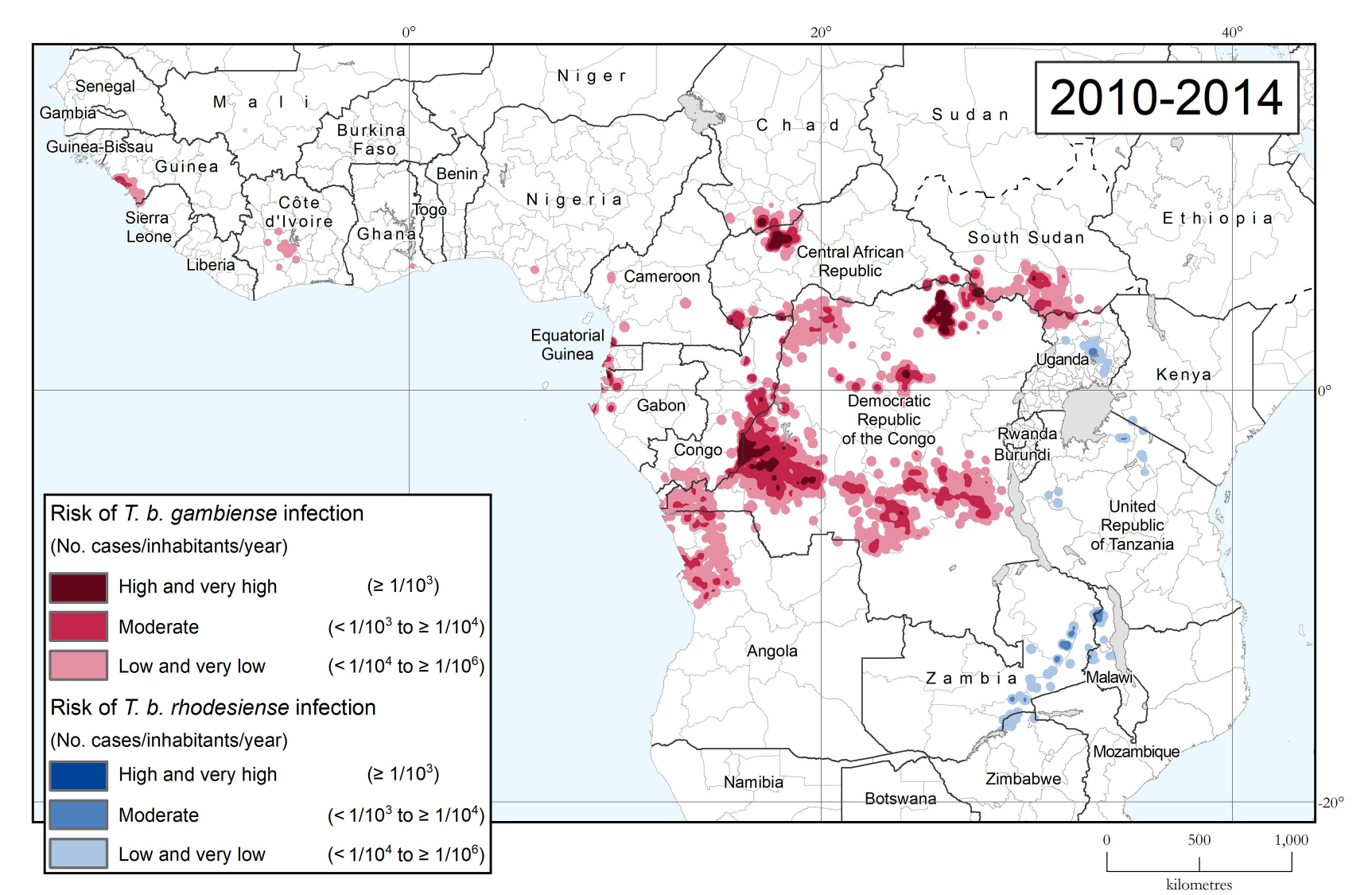

## Slide 12
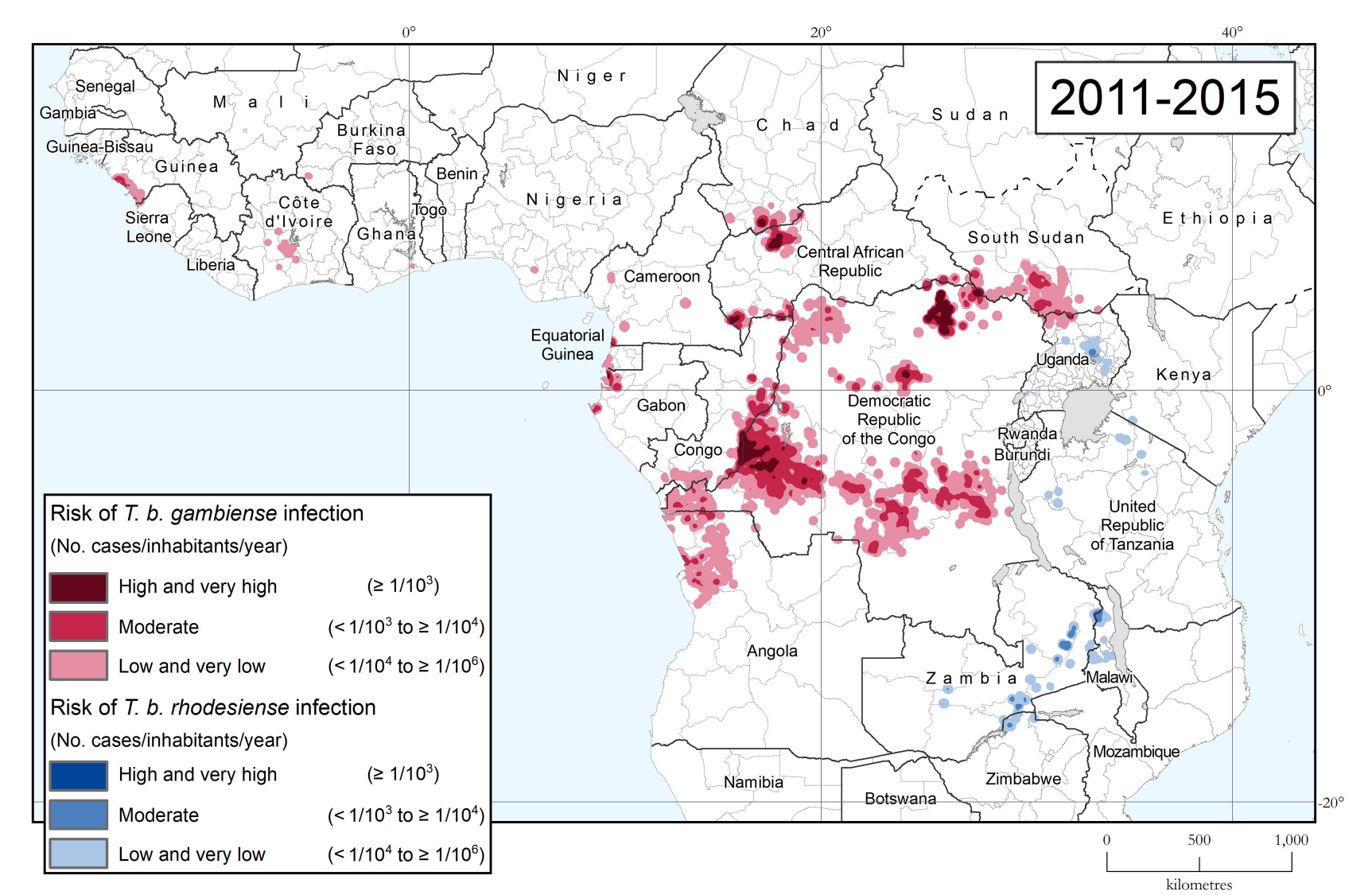

## Slide 13
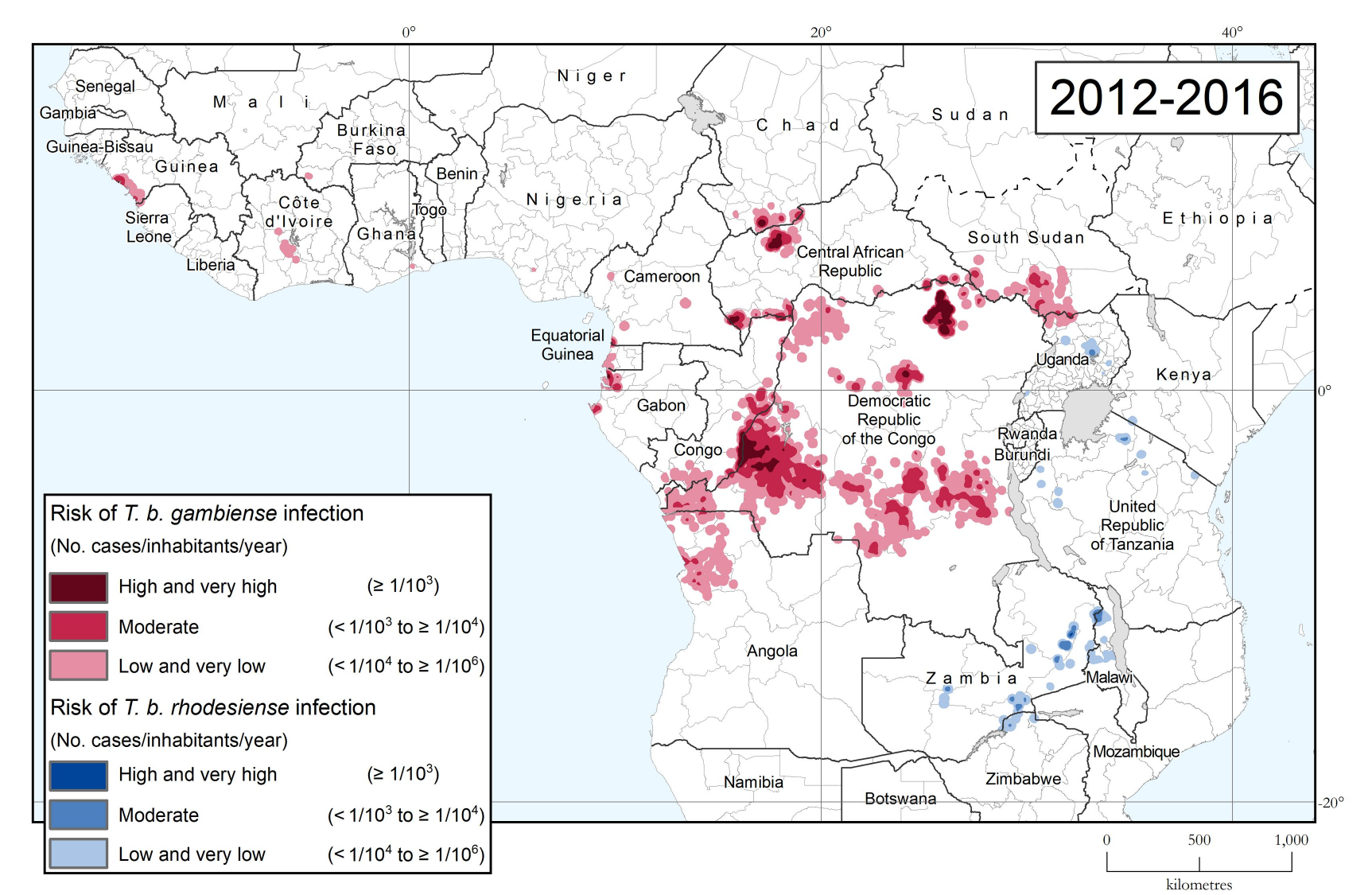

## Slide 14
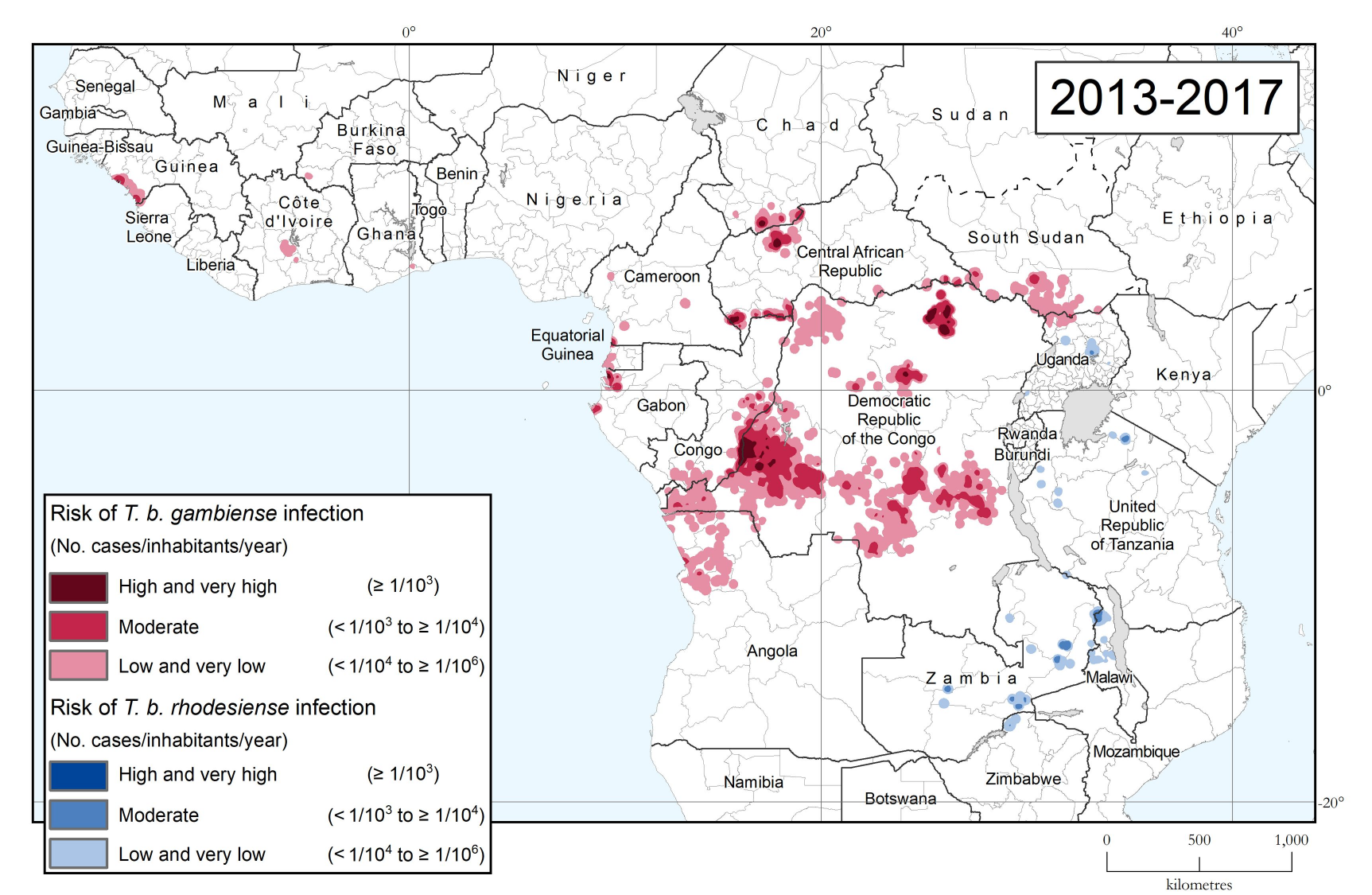

## Slide 15
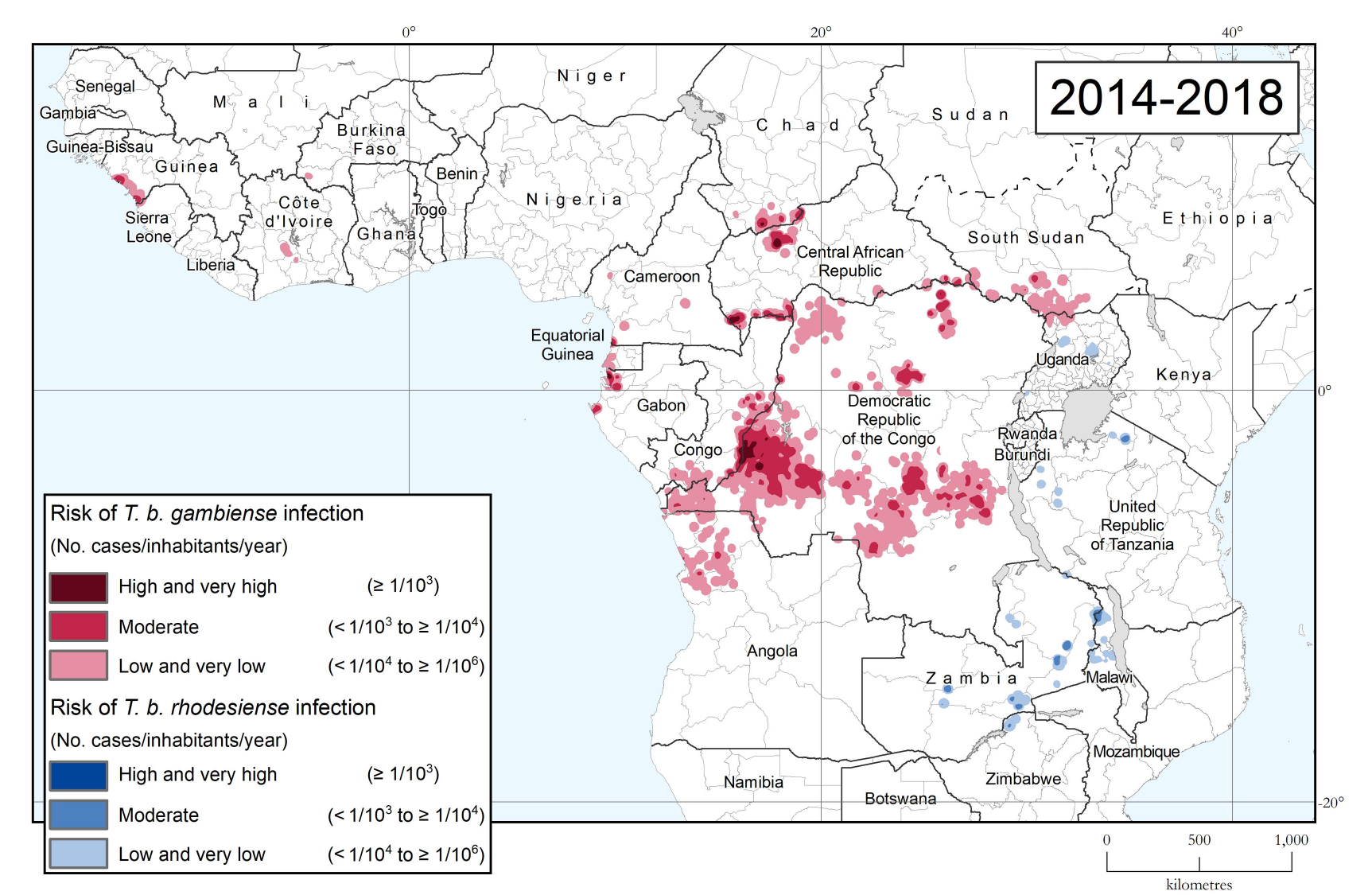

## Slide 16
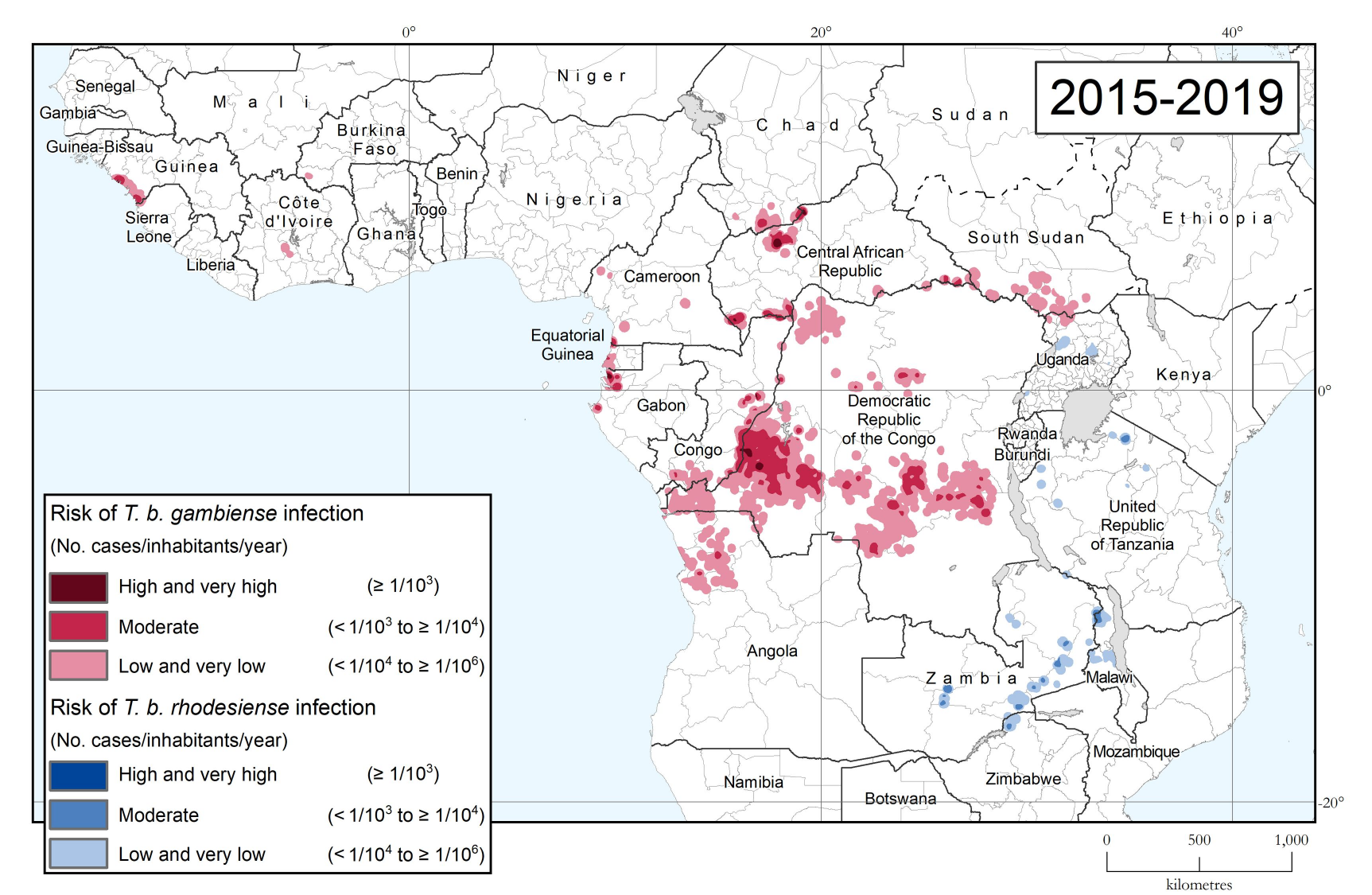

## Slide 17
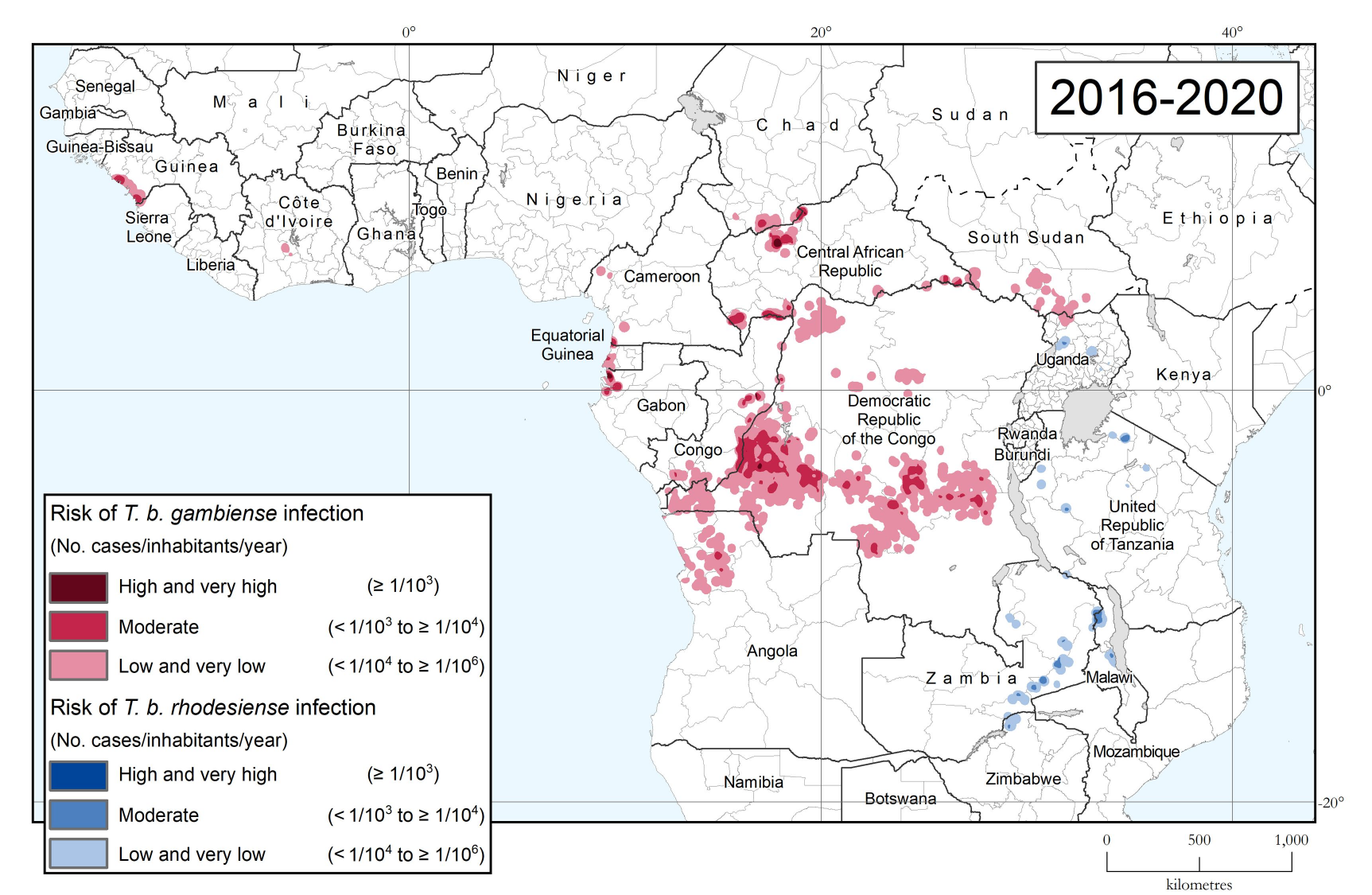

Supplement: S4 File — The base layers used in the maps are the FAO Global Administrative Unit Layers (GAUL) and FAO Inland water bodies in Africa. (PPSX) [file pntd.0010047.s004.ppsx]
