# Supplementary material for: The elimination of human African trypanosomiasis: Achievements in relation to WHO road map targets for 2020
Source: PLoS Negl Trop Dis. 2022 Jan 18;16(1):e0010047. doi: 10.1371/journal.pntd.0010047 (PMC8765662; doi:10.1371/journal.pntd.0010047)
Supplement: S5 File — Period 2016–2020 (by country). (DOCX) [file pntd.0010047.s005.docx]

# Population at risk of gambiense and rhodesiense HAT

Table 1 Population at risk of *T. b. gambiense* infection (no. persons × 10^3^). Period 2016–2020.

| **Country** | **Total country population**  **2020*** | **Population at risk**  **2016-2020** | | | | | |
| --- | --- | --- | --- | --- | --- | --- | --- |
|  |  | **Very High**  **and High** | **Moderate** | **Low and**  **Very Low** | **Total**  **at risk** | **% of total**  **country**  **population** |  |
| Angola | 32,522 | 0 | 41 | 1,994 | 2,035 | 6.3 |  |
| Burkina Faso | 20,833 | 0 | 0 | 0 | 0 | 0 |  |
| Cameroon | 27,744 | 0 | 37 | 200 | 236 | 0.9 |  |
| Central African Republic | 5,263 | 8 | 153 | 581 | 741 | 14.1 |  |
| Chad | 16,876 | 0 | 47 | 568 | 615 | 3.6 |  |
| Congo | 5,293 | 0 | 34 | 1,480 | 1,514 | 28.6 |  |
| Côte d'Ivoire | 27,470 | 0 | 0 | 279 | 279 | 1.0 |  |
| Democratic Republic of the Congo | 101,769 | 4 | 2,471 | 40,265 | 42,740 | 42.0 |  |
| Equatorial Guinea | 837 | 0 | 15 | 36 | 51 | 6.1 |  |
| Gabon | 2,230 | 2 | 13 | 27 | 41 | 1.9 |  |
| Guinea | 12,527 | 0 | 158 | 2,352 | 2,510 | 20.0 |  |
| Sierra Leone | 6,644 | 0 | 1 | 188 | 189 | 2.9 |  |
| South Sudan | 10,561 | 0 | 0 | 1,051 | 1,051 | 10.0 |  |
| Uganda | 43,250 | 0 | 0 | 423 | 423 | 1.0 |  |
| Other Endemic Countries** | 333,783 | 0 | 0 | 0 | 0 | 0 |  |
| Total | 647,601 | 13 | 2,969 | 49,443 | 52,425 | 8.1 |  |

* As per Landscan

** Countries at marginal risk: Benin, Gambia, Ghana, Guinea-Bissau, Liberia, Mali, Niger, Nigeria, Senegal and Togo.

Table 2 Population at risk of *T. b. rhodesiense* infection (no. persons × 10^3^). Period 2016–2020.

| **Country** | **Total country population**  **2020*** | **Population at risk**  **2016-2020** | | | | |
| --- | --- | --- | --- | --- | --- | --- |
|  |  | **Very High**  **and High** | **Moderate** | **Low and**  **Very Low** | **Total**  **at risk** | **% of total**  **country**  **population** |
| Malawi | 19,823 | 0 | 126 | 775 | 901 | 4.5 |
| United Republic of Tanzania | 60,363 | 0 | 2 | 306 | 308 | 0.5 |
| Uganda | 43,250 | 0 | 0 | 805 | 805 | 1.9 |
| Zambia | 18,523 | 0 | 13 | 464 | 477 | 2.6 |
| Zimbabwe | 14,546 | 0 | 0 | 9 | 9 | 0.1 |
| Other Endemic Countries** | 222,326 | 0 | 0 | 0 | 0 | 0 |
| Total | 378,831 | 0 | 141 | 2,359 | 2,500 | 0.7 |

* As per Landscan

** Countries at marginal risk: Botswana, Burundi, Eswatini, Ethiopia, Kenya, Mozambique, Namibia and Rwanda.
