# Supplementary material for: The elimination of human African trypanosomiasis: Achievements in relation to WHO road map targets for 2020
Source: PLoS Negl Trop Dis. 2022 Jan 18;16(1):e0010047. doi: 10.1371/journal.pntd.0010047 (PMC8765662; doi:10.1371/journal.pntd.0010047)
Supplement: S6 File — (DOCX) [file pntd.0010047.s006.docx]

People at risk of HAT that are potentially covered by facilities with diagnostic and treatment capabilities for HAT.

Table 1 People at risk of HAT that are potentially covered by facilities with diagnostic and treatment capabilities for HAT

| **Risk category** | **People at risk** | **People at risk covered by facilities with HAT capabilities** | | | | | | | | | | | |
| --- | --- | --- | --- | --- | --- | --- | --- | --- | --- | --- | --- | --- | --- |
|  |  | **Diagnosis** | | | | | | **Treatment** | | | | | |
|  |  | **≤ 1-hour travel** | | **≤ 3-hour travel** | | **≤ 5-hour travel** | | **≤ 1-hour travel** | | **≤ 3-hour travel** | | **≤ 5-hour travel** | |
|  | (no. persons × 10^3^) | (no. persons × 10^3^) | % of at risk | (no. persons × 10^3^) | % of at risk | (no. persons × 10^3^) | % of at risk | (no. persons × 10^3^) | % of at risk | (no. persons × 10^3^) | % of at risk | (no. persons × 10^3^) | % of at risk |
| **gambiense HAT** | | | | | | | | | | | | | |
| High and very high | 13 | 8 | 59 | 9 | 71 | 10 | 76 | 8 | 59 | 9 | 71 | 10 | 76 |
| Moderate | 2,969 | 1,362 | 46 | 2,376 | 80 | 2,708 | 91 | 1,349 | 45 | 2,337 | 79 | 2,697 | 91 |
| Low and very low | 49,443 | 31,262 | 63 | 40,825 | 83 | 45,377 | 92 | 25,661 | 52 | 39,078 | 79 | 44,388 | 90 |
| Total | 52,425 | 32,632 | 62 | 43,211 | 82 | 48,096 | 92 | 27,018 | 52 | 41,426 | 79 | 47,096 | 90 |
| **rhodesiense HAT** | | | | | | | | | | | | | |
| High and very high | - | - | - | - | - | - | - | - | - | - | - | - | - |
| Moderate | 141 | 45 | 32 | 106 | 75 | 125 | 89 | 15 | 11 | 68 | 48 | 101 | 71 |
| Low and very low | 2,359 | 711 | 30 | 1,536 | 65 | 1,949 | 83 | 517 | 22 | 1,270 | 54 | 1,770 | 75 |
| Total | 2,500 | 756 | 30 | 1,642 | 66 | 2,074 | 83 | 532 | 21 | 1,338 | 54 | 1,871 | 75 |
